# Integrating Artificial Intelligence (AI) in Primary Health Care (PHC) Systems: A Framework-Guided Comparative Qualitative Study

**DOI:** 10.3390/healthcare14020145

**Published:** 2026-01-07

**Authors:** Farzaneh Yousefi, Reza Dehnavieh, Maude Laberge, AliAkbar Haghdoost, Maxime Sasseville, Somayeh Noori Hekmat, Mohammad Mehdi Ghaemi, Mohsen Nadali

**Affiliations:** 1 Faculty of Nursing, Université Laval, Pavillon Ferdinand-Vandry, Quebec City, QC G1V 0A6, Canada; farzaneh.yousefi.1@ulaval.ca (F.Y.);; 2 Department of Management, Policy and Health Economics, Faculty of Medical Information and Management, Kerman University of Medical Sciences, Kerman 76169-13555, Iran; 3 Health Foresight and Innovation Research Center, Institute for Futures Studies in Health, Kerman University of Medical Sciences, Kerman 76169-13555, Iran; 4 Faculté de Médecine, Université Laval, Pavillon Ferdinand-Vandry, Quebec City, QC G1V 0A6, Canada; 5 Centre de Recherche du CHU de Quebec–Université Laval (CRCHUQ), CHU de Québec–Université Laval, Quebec City, QC G1E 6W2, Canada; 6 VITAM Centre de Recherche en Santé Durable, Quebec City, QC G1J 2G1, Canada; 7 HIV/STI Surveillance Research Center, and WHO Collaborating Center for HIV Surveillance, Institute for Futures Studies in Health, Kerman University of Medical Sciences, Kerman 76169-13555, Iran; 8 Medical Informatics Research Center, Institute for Futures Studies in Health, Kerman University of Medical Sciences, Kerman 76169-13555, Iran; 9 Department of Business Administration, Faculty of Management, Mehr Alborz University, Tehran 31987-64653, Iran

**Keywords:** artificial intelligence (AI), Primary Health Care (PHC), AI in PHC, expert perspectives, qualitative study, comparative study, interview, focus group

## Abstract

**Highlights:**

**What are the main findings?**

- AI integration in PHC depends primarily on systemic alignment across governance, financing, infrastructure, and workforce domains rather than technology alone.
- Quebec and Iran demonstrate distinct AI integration trajectories shaped by differences in system maturity and institutional capacity.

**What are the implications of the main findings?**

- Strengthening governance coordination, sustainable financing mechanisms, and interdisciplinary workforce development are central to successful AI implementation in PHC.
- Comparative insights underscore the need for phased, context-sensitive digital transformation strategies rather than one-size-fits-all approaches.

**Abstract:**

**Background/Objectives**: The integration of artificial intelligence (AI) into primary health care (PHC) holds significant potential to enhance efficiency, equity, and clinical decision-making. However, its implementation remains uneven across contexts. This study aimed to identify the systemic, contextual, and governance-related determinants influencing AI readiness in PHC, comparing two distinct health systems, Quebec (Canada) and Iran. **Methods**: A qualitative, comparative design was employed. Data were collected through semi-structured interviews and focus group discussions with key informants in both settings. A framework-guided content analysis was conducted based on the four Primary Care Evaluation Tool (PCET): stewardship, financing, resource generation, and service delivery. The analysis explored shared context-specific challenges and requirements for AI implementation in PHC. **Results**: Analysis revealed that AI readiness is shaped more by systemic coherence rather than technological availability alone. Across both contexts, governance- and financing-related challenges were reported by the majority of participants, alongside limited data interoperability. In Quebec, challenges were more commonly articulated around operational and ethical concerns, including workflow integration, transparency, and professional trust. In contrast, participants in Iran emphasized foundational deficiencies in governance stability, financing mechanisms, and digital infrastructure as primary barriers. Across both settings, adaptive governance, sustainable investment, data standardization, and workforce capacity-building consistently emerged as key requirements for AI integration in PHC. **Conclusions**: AI readiness in PHC is a multidimensional process, in which implementation priorities must align with system maturity. This comparative analysis underscores that while high-resource systems must prioritize ethical integration and workflow alignment, middle-resource settings require foundational investments in governance and infrastructure. This reinforces that AI readiness is a context-dependent and phased process rather than a one-size-fits-all endeavor.

## 1. Introduction

In recent years, artificial intelligence (AI) has emerged as a central driver of digital transformation in healthcare, attracting substantial attention from researchers, policymakers, and industry leaders [1,2]. Global investment in AI for health increased from USD 1.1 billion in 2016 to USD 22.4 billion in 2023, with projections exceeding USD 208 billion by 2030 [3,4]. AI refers broadly to computational systems capable of performing tasks traditionally associated with human cognition, including reasoning, learning, pattern recognition, and natural language understanding. These capabilities have enabled multiple clinical and operational applications, from predictive analytics and automated triage to natural language processing and computer vision for diagnostic imaging [5,6]. Despite the rapid expansion of these innovations across different levels of care, the integration of AI within Primary Health Care (PHC), the front line of most health systems, remains comparatively underexplored [7,8,9].

PHC represents a critical domain for AI deployment, given its role in promoting access, continuity, prevention, and community clinical decision-making, facilitating remote monitoring, and streamlined administrative processes [8,10,11]. Yet, integrating such technologies into PHC is profoundly complex. Barriers frequently arise from systemic rather than purely technical constraints, including gaps in governance and regulatory frameworks, inadequate digital infrastructure, fragmented or immature data-governance arrangements, limited interoperability across health information systems, and persistent ethical concerns related to transparency, bias, and privacy [10,12]. Addressing these barriers requires coordinated efforts in governance, regulation, workforce development, and sustainable financing, areas where PHC systems differ substantially in their institutional maturity and resource environments [10].

Although challenges to AI integration in PHC are observed worldwide, their manifestations and potential solutions vary significantly across contexts. Differences in digital maturity, organizational structures, and socio-economic conditions shape both the nature and severity of barriers. High-resource settings such as Quebec, where digital health infrastructure is relatively advanced, tend to grapple with challenges related to integrating AI tools into established platforms, ensuring interoperability across institutions, and maintaining public trust in data-driven decision-making [13,14]. In response, these systems often emphasize expanding interoperability standards, strengthening ethical oversight, and increasing stakeholder engagement in AI governance [13,15]. Conversely, in resource-constrained contexts such as Iran, barriers are more foundational and include fragmented governance [16,17], the lack of a unified national AI strategy for PHC [18], limited digital infrastructure in rural or underserved regions [19], and shortages of professionals trained in AI and digital health [20]. Addressing these gaps requires building coherent governance structures, investing in infrastructure, and fostering multi-sectoral collaboration to develop both technical and human capacity [21,22].

These contrasting realities underscore the value of a comparative inquiry. Examining AI integration in PHC across one high-resource, digitally mature system and another resource-constrained system experiencing early-stage digital transformation provides an opportunity to identify cross-cutting challenges alongside context-specific pathways for action. Quebec’s experience with large-scale digital health initiatives, such as provincial telehealth platforms and AI-assisted triage tools, offers practical insights [13,23] that may inform strategic planning in settings with emerging infrastructures. Meanwhile, Iran’s long-standing community-based PHC network [24], anchored by health houses and rural health workers [25,26], represents a service delivery model with strong potential for equity-oriented, locally adapted AI applications [25]. Comparative analyses of this kind yield evidence to support tailored policy development, inform resource allocation, and enhance international knowledge exchange on AI readiness in PHC.

Despite growing scholarly attention to AI in PHC, including systematic reviews, pilot projects, and qualitative studies [27,28], most investigations remain confined to single-country contexts. Consequently, there is limited understanding of how systemic and contextual determinants of AI integration compare across diverse health system environments. Without such evidence, policymakers risk adopting strategies that may be effective elsewhere but poorly aligned with local priorities and organizational capacities, potentially resulting in inefficiencies or amplifying inequities. To address this gap, the present study undertakes a comparative qualitative analysis of expert perspectives from Quebec and Iran. Specifically, the study asks: (1) What common and context-specific barriers and enablers shape AI integration in PHC across these two health systems? (2) To what extent are experiences and lessons transferable between high- and middle-resource settings? (3) What are the implications of these findings for equity-oriented policy planning and AI readiness in PHC? By examining these issues through the perspectives of actors directly involved in PHC governance, digital health initiatives, and service delivery, this study offers a deeper understanding of the systemic and contextual factors that shape AI integration in real-world settings.

## 2. Materials and Methods

### 2.1. Study Design

We conducted a comparative qualitative study using a multiple-case (two-case) design to explore the challenges and requirements for implementing AI in PHC across two contrasting contexts. Grounded in an interpretivist paradigm with a pragmatic orientation, this design was particularly suited to capturing participants’ perspectives within their socio-cultural and institutional settings, while generating findings with practical direct relevance for policy and practice [29,30]. Because decisions around the design, adoption, and use of AI are fundamentally shaped by human cognition, professional judgment, and socio-technical interactions, a qualitative approach was essential for gaining insight into how stakeholders interpret, negotiate, and engage with AI in real-world PHC settings [31,32].

The unit of analysis was the national PHC system, with each case (Quebec and Iran) representing a distinct PHC system deliberately chosen for their contrasting levels of digital maturity and resource availability. Within each case, key informants with recognized expertise in PHC and AI were purposively recruited to provide diverse and contextually grounded insights.

Reporting of the study follows the Consolidated Criteria for Reporting Qualitative Research (COREQ) [33]. A completed COREQ checklist indicating the location of each reporting criterion within the manuscript is provided as Supplementary Material S1.

### 2.2. Settings

This study was conducted in two distinct health system contexts: Quebec, Canada, and Iran. In Quebec, PHC is primarily organized around family medicine groups (groupes de médecine de famille, FMGs) and community health centers, with the provincial government overseeing financing, regulation, and service coordination [34,35]. The system benefits from universal health coverage and a mature digital health infrastructure, including province-wide electronic health record (EHR) systems and telehealth platforms [36]. In recent years, Quebec has invested in digitally enabled primary care, including remote patient monitoring (telemonitoring) programs and virtual care/eConsult services in PHC; early explorations relevant to AI-enabled triage and population-level analytics are also emerging within Canadian primary care initiatives [37,38,39].

In Iran, PHC is delivered through an extensive national health network that integrates rural health houses, urban and rural health centers, and comprehensive community health centers. The system is grounded in a strong community-based approach, with trained health workers (Behvarzes) and family physicians serving as the first point of contact [40,41]. While digital health initiatives have expanded over the past decade, including the development of national electronic health record systems and pilot AI projects, implementation in PHC remains at an early stage, particularly in rural regions [24,42].

### 2.3. Participants and Recruitment

Participants were recruited in both Quebec and Iran using purposive sampling, supplemented by a snowball approach to capture diverse perspectives. Recruitment targeted three main groups: (1) health managers and directors within the PHC system; (2) frontline PHC providers such as family physicians and community health workers; (3) experts and academics in digital health and AI with recognized relevance to PHC. They, who had no prior relationship with the research team, were identified through professional networks and academic affiliations and were invited by direct email contact.

While recruitment in Iran aimed to include frontline community health workers (Behvarzes), who are predominantly female, practical access constraints and reliance on institutional gatekeepers resulted in a final sample primarily composed of individuals in managerial, policy, and academic roles. This sampling outcome, including the absence of female Behvarzes’ perspectives, is acknowledged and critically reflected upon as a limitation in the Discussion (Section 4.3).

Eligibility criteria required participants to be ≥18 years of age, with at least one year of professional experience in PHC or AI-related health fields, and to occupy roles relevant to clinical practice, management, policy, or academia. These criteria were designed to ensure that participants had direct exposure to PHC decision-making processes and the organizational realities of digital health initiatives. Given the structural and organizational differences between the two PHC systems, eligibility criteria were conceptually aligned but contextually adapted in each setting. Table 1 summarizes the inclusion and exclusion criteria applied in Quebec and Iran and illustrates how these contextual adaptations informed participant selection within the comparative design.

While the study did not aim to examine the effects of demographic or professional characteristics on AI readiness, differences in the composition of the two samples should be considered when interpreting the comparative findings. These differences may have influenced how AI-related challenges and priorities were articulated across contexts.

Recruitment and data collection were conducted iteratively, and the final number of participants in each context was determined by thematic saturation and information power rather than by predefined numerical targets. Interviews continued until no substantively new themes emerged within each setting and sufficient depth was achieved across the identified stakeholder groups. Minor differences in participant numbers between Quebec and Iran reflected contextual and pragmatic considerations, including differences in health system structure, stakeholder availability, and organizational roles within PHC, while maintaining analytical comparability across cases.

### 2.4. Participant Characteristics

Understanding participants’ backgrounds helped contextualize their perspectives on AI implementation within PHC. The demographic and professional characteristics of participants in Quebec and Iran are summarized in Table 2. Because participants’ professional profiles and organizational structures differed across the two settings, not all categories were directly comparable. These distinctions reflect the contextual nature of each PHC system rather than inconsistencies in coding.

A total of 15 participants from Quebec and 16 from Iran took part in the study. The Quebec sample included both men and women with a balanced distribution, while all Iranian participants were male. Most participants in both contexts were between 45 and 64 years of age, indicating senior professional status. Participants in Iran tended to have longer professional experience, with nearly all reporting more than 16 years of practice, whereas the Quebec sample included a broader mix of early- and late-career professionals. Educational level backgrounds were highly advanced in both groups. Nearly three-quarters of participants in Quebec and almost all in Iran held doctoral degrees, reflecting strong academic and research profiles.

The two samples differed in disciplinary composition. Quebec participants represented diverse fields, computer science and AI, public health, neuroscience, digital health, and biomedical engineering, while Iranian participants were mainly from disciplines directly related to PHC, including family medicine, health foresight, health policy and management, and health IT. These disciplinary differences were reflected in participants’ professional roles: Participants from Quebec were primarily drawn from technical, academic, and innovation-oriented positions, whereas the Iranian sample mainly included individuals in managerial, planning, and PHC administrative roles. Institutional affiliations reflected these structural distinctions. Participants from Quebec were mainly affiliated with Université Laval, McGill University, and Mila Institute for AI, while Iranian participants were linked to medical universities (Tehran, Kerman, Isfahan), the Ministry of Health and Medical Education, health insurance organizations, and professional associations.

### 2.5. Ethical Considerations

Ethical approval for the Quebec study was obtained from the Research Ethics Board of Université Laval (approval code: 2023-443), covering the period from February 2024 to March 2025. Preparatory activities, including the development and adaptation of interview guides, were conducted during the fall and winter of 2023. In Iran, ethical approval was granted by the Research Ethics Committee of Kerman University of Medical Sciences (approval code: IR.KMU.REC.1402.182) in September 2023. In both settings, participants received an information sheet outlining the study objectives, interview procedures, and confidentiality measures, and provided informed consent before participation. All data were anonymized and stored securely in accordance with institutional and national guidelines. All procedures complied with the ethical standards of the Declaration of Helsinki.

### 2.6. Data Collection

Data were gathered through semi-structured interviews in both Quebec and Iran, complemented by one focus group discussion in Iran. An interview guide, developed collaboratively by the research team and shared with participants in advance, explored experiences with PHC systems, perceived challenges, and requirements for AI integration across stewardship, financing, resource generation, and service delivery. The full semi-structured interview script is provided in Supplementary Material S2.

To ensure a shared understanding, AI was introduced to participants using a broad, functional definition. In both the English and Persian interview guides, AI was described as encompassing any digital system, application, or tool that uses data-driven or algorithmic techniques to support decision-making, automation, prediction, or service delivery in PHC. This intentionally inclusive framing was adopted to capture a wide range of experiences with AI-enabled and intelligent digital systems across diverse contexts.

In Quebec, interviews were conducted between April and August 2024 with PHC experts and stakeholders. Most were carried out online via Microsoft Teams (Microsoft Corporation, Redmond, WA, USA), in either English or French, depending on participant preference. To accommodate participants unable to attend synchronously, written responses were also accepted through an online questionnaire.

In Iran, interviews took place between May 2024 and March 2025, conducted online via Google Meet (Google LLC, Mountain View, CA, USA) or Skyroom (Mahbang Technology Pars, Iran) in Persian. As in Quebec, some participants provided written responses through an online platform (System Gostar Chista Co., Hamedan, Iran)). Additionally, in Iran, one focus group discussion was added to complement the individual interviews. This decision was driven by practical and contextual considerations. In the Iranian PHC system, key stakeholders involved in AI-related activities often operate across multiple administrative layers and are typically available through coordinated group meetings rather than individual sessions. Additionally, collective discussions tend to facilitate open dialogue, reduce the formality associated with one-on-one interviews, and allow participants to reflect on shared challenges and interdependent responsibilities within the PHC network. The focus group, therefore, provided an efficient and contextually appropriate means to capture perspectives that could not be fully accessed through individual interviews alone.

Across both settings, all interviews and the focus group were audio-recorded and transcribed verbatim with participant consent. A web-based platform speech-to-text tool (dictation.io; Digital Inspiration, India) was used to support the initial transcription process, followed by repeated manual review and correction through audio playback to ensure accuracy and completeness. Written responses were integrated into the dataset, and supplementary field notes were maintained to document contextual observations. Data collection in both Quebec and Iran was conducted by the primary researcher (PhD candidate), who was responsible for participant recruitment, conducting all interviews and the focus group, and leading the data analysis. Supervisory support was provided by different academic advisors in each context; however, they were not involved in direct data collection. This approach ensured methodological consistency across settings and supported comparability between cases.

### 2.7. Data Analysis

All interview transcripts, focus group notes, and written responses were analyzed using a framework-themes [43,44] approach guided by the Primary Care Evaluation Tool (PCET). The analysis followed several iterative steps. First, transcripts and written responses were reviewed line by line, and initial codes were generated inductively from participants’ quotations. Two researchers independently coded the data and compared their outputs, resolving discrepancies through discussion until consensus was achieved [45].

**Figure 1:**
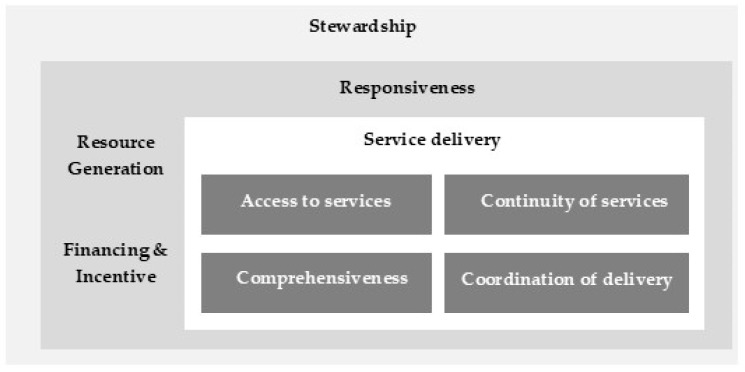
The framework of the Primary Care Evaluation Tool (PCET) [46].

Next, codes were organized deductively within the four dimensions of the PCET framework. As illustrated in Figure 1, the PCET framework is composed of four core dimensions (stewardship, financing and incentives, resource generation, and service delivery) as well as a set of sub-dimensions related to accessibility, continuity, comprehensiveness, and coordination of services [46]. The figure visually represents how these dimensions intersect to capture both organizational functions and performance outcomes within PHC systems and served as the analytical map guiding our deductive coding.

The PCET was selected because it provides a comprehensive and internationally recognized framework for assessing the organization and performance of PHC systems, aligning well with the study’s objective to identify governance, financial, resource-generation, and service-delivery requirements for AI integration. Within each dimension, codes were further clustered into main themes and subthemes, reflecting progressively higher levels of abstraction. This process allowed both emergent insights and framework-driven organization to be integrated coherently.

Data analysis was conducted manually using Microsoft Excel (Microsoft Corporation, Redmond, WA, USA) to systematically code, categorize, and compare data across cases. This method enhanced transparency and traceability of the analytic process without relying on specialized software such as NVivo or MAXQDA.

Finally, a cross-case analysis was conducted to systematically compare findings between Quebec and Iran, following established approaches for cross-case displays and pattern identification [47,48]. After identifying challenges and requirements for each country within the four PCET dimensions, a three-column comparative matrix was developed to align the main and sub-themes across both contexts. Themes that appeared in both countries were placed in the central column as shared (convergent) issues, while those specific to Quebec and Iran were positioned in the left and right columns, respectively. This visual comparison facilitated the identification of common patterns and context-specific factors, which were subsequently illustrated in Venn diagrams to demonstrate thematic convergence and divergence across settings. This comparative synthesis enabled a nuanced understanding of how variations in governance structures, digital maturity, and resource environments influenced pathways for AI implementation in PHC.

### 2.8. Credibility and Trustworthiness of the Data

Multiple strategies were applied to ensure the credibility and trustworthiness of the findings, consistent with established criteria for qualitative research [45]. Credibility was supported by prolonged engagement with participants across two countries, the use of multiple data sources, and iterative discussion between two researchers during coding to reach consensus. Informal peer debriefing within the research team further enhanced interpretive rigor. Dependability was ensured through an explicit audit trail of coding decisions, theme development, and analytic reflections systematically maintained in Microsoft Excel to enhance traceability. Confirmability was promoted through reflexive discussions among the research team to minimize individual bias and ensure that interpretations were grounded in participants’ accounts. Transferability was facilitated by providing detailed contextual descriptions of the PHC systems in Quebec and Iran, allowing readers to assess the relevance and applicability of the findings to other health system settings.

## 3. Results

The findings of the comparative qualitative study on the implementation of AI in PHC in Quebec and Iran are presented in two parts. First, country-specific findings are reported, focusing on the challenges and requirements identified in each setting. Second, a cross-case comparative analysis is conducted, structured around the four dimensions of the PCET framework.

### 3.1. Country-Specific Findings

This section presents the country-specific results structured according to the PCET framework. For each setting, challenges and requirements related to the implementation of AI in PHC are summarized in tables and further explained through narrative synthesis. The tables display the main themes and sub-themes identified under each PCET dimension, while the accompanying text elaborates on their implications and provides illustrative evidence where relevant. Findings from Quebec are presented first, followed by those from Iran, to highlight both contextual differences and points of convergence in AI readiness and implementation pathways.

#### 3.1.1. Challenges and Requirements of AI Implementation in PHC: Insights from Quebec

In Quebec, analysis of challenges yielded 133 codes, organized into 34 sub-themes and 12 main themes across the four PCET dimensions. In parallel, the analysis of requirements produced 144 codes, grouped into 29 sub-themes and 10 main themes across three PCET dimensions (stewardship, financing and incentives, and resource generation). Together, these analyses reveal a complex and multidimensional landscape of both barriers and enabling conditions shaping the implementation of AI in PHC. Table 3 provides a structured overview of these themes, while the detailed categorizations and participant quotes are presented in Supplementary Material S3, Tables S1 and S2.

- Stewardship

Consistently highlighted governance-related issues as one of the most critical determinants of AI implementation in Quebec’s PHC system. A recurrent theme concerned the misalignments between technological solutions and the realities of primary health care practice, noting that innovations often failed to meet the needs of either patients or professionals. As one respondent explained, *“Even if an application is excellent, if it is unusable for physicians, it will not be adopted”* (P10). The absence of a comprehensive strategy was another recurring challenge, leaving many initiatives as *“small islands with no overarching vision”* (P11). In addition, slow approval processes and constantly changing regulatory frameworks hindered system-wide integration. To address these issues, participants called for the development of patient-centered and supportive policies, emphasizing that *“AI is a tool that facilitates the management of care and services… it serves humans and cannot replace them”* (P4). Strengthening leadership, intersectoral collaboration, and transparent regulations were seen as crucial for building public trust and ensuring accountability in AI use.

- Financing & incentives

Financial constraints emerged as a major barrier. Respondents described inefficiencies in securing resources and the high costs of development and approval, with one noting, “*Obtaining approval for a medical device from Health Canada costs between 3 to 5 million dollars… and even after that, no sales have yet been made*” (P13). Funding shortages often forced projects to pause, while reimbursement systems were perceived as outdated and misaligned with AI-based services. To move forward, participants proposed creating dedicated funding streams and more rapid evaluation mechanisms. They also underlined the importance of cost–benefit assessments before implementation, with one emphasizing, “*New AI tools must be evaluated for cost–benefit before implementation*” (P15)

- Resource generation

Challenges in infrastructure and resources were recurrent. Participants pointed to obsolete IT systems, interoperability gaps, and limited tools for virtual care, which complicated AI integration. For instance, one physician explained, “*The patient’s device was not compatible with my EMR system, which required manual data integration*” (P12). Concerns about cybersecurity and fragmented data systems further complicated the use of AI, with risks of inaccuracy in clinical decision-making. Requirements focused on building sustainable and interoperable infrastructures, strengthening clinical research through diverse datasets, and ensuring accurate, transparent data management. Participants also stressed the need for workforce training and interdisciplinary capacity-building: “*We need bilingual experts who understand both AI and the clinical domain to bridge this gap*” (P10).

- Service delivery

At the delivery level, concerns centered on workload, equity, and the patient–provider relationship. Some highlighted risks of digital divides: “*Some communities may not have equal access to AI tools, leading to inequalities in health services*” (P15). Others feared that technology could undermine the human dimension of care, with one warning that “*this technology will be seen only as a tool for saving time and resources, rather than truly improving the system*” (P14). Overall, these views reflected a cautious attitude toward AI’s role in service delivery, emphasizing that without adequate support and inclusiveness, digital transformation could unintentionally deepen disparities and weaken the relational foundations of primary care.

#### 3.1.2. Challenges and Requirements of AI Implementation in PHC: Insights from Iran

In Iran, analysis of challenges yielded 172 codes, organized into 37 sub-themes and 11 main themes across the four PCET dimensions. In parallel, the analysis of requirements produced 156 codes, grouped into 32 sub-themes and 9 main themes. These findings highlighted systemic, financial, infrastructural, and cultural barriers to the implementation of AI in PHC, alongside concrete requirements identified by participants. Table 4 provides a detailed synthesis of these challenges and requirements, while the detailed analysis of participants’ perspectives is presented in Supplementary Material S3, Tables S3 and S4.

- Stewardship

Participants consistently reported the absence of a national AI strategy, managerial instability, and fragmented coordination as core governance barriers. Several also warned about impulsive decision-making and weak governance: “*I find this issue dangerous… many actions in our country have not reached desirable outcomes because they were impulsive and poorly planned.*” (P25); “*There is no national coordination… issues like data confidentiality are not addressed—if we don’t process our own data, foreign actors will do it for us.*” (P20). Regulatory rigidity further constrained progress: “*With the current regulations, it is impossible to carry out AI projects in this country.*” (P22). Requirements focused on developing a comprehensive national strategy and roadmap, creating coordinating structures (e.g., an AI Council), and establishing clear legal-ethical frameworks with human oversight. As respondents put it: “*A national strategy must be developed for AI use… with clear goals, regulatory mechanisms, and continuous monitoring.*” (P25); “*If I were the Minister of Health, I would establish an AI Council in the Ministry.*” (P27); “*Systems to verify AI performance and precise standards for evaluating its effectiveness are essential…*”; “*There must always be a human supervisor engaged*” (P20; P19).

- Financing & incentives

Stakeholders highlighted limited and inefficient resource allocation, high up-front costs, low willingness to invest, and misaligned payment mechanisms as major barriers. For instance: “*Funding depends heavily on scale; it can range from 500 million tomans (approximately USD 10,000) up to 5–6 billion tomans (approximately USD 100,000–120,000) if we bring in national datasets.*” (P18); “*Insurers are reluctant to invest in preventive services, fearing that longer life expectancy will increase long-term costs.*” (P28); and “*The tariff system has not introduced AI-specific codes.*” (P24). Proposed requirements included sustainable investment and payment models aligned with AI-enabled services, such as mixed or context-specific schemes: “*We need to design a hybrid model, something like a mix of fixed payments and performance-based incentives.*” (P21); “*In communities with fewer than 200,000 residents, we may be able to design and test a new payment model… for underserved regions.*” (P29); “*alongside large-scale investment in AI equipment*” (P26).

- Resource generation

Participants emphasized immature digital infrastructure, poor interoperability, fragmented/low-quality data, restricted access, security risks, and shortages of AI-literate human resources. Illustratively: “*Even at top universities in Tehran, IT infrastructure is insufficient.*” (P27); “*Different provinces and counties develop their own apps, but these AI systems cannot interact or exchange data.*” (P21); “*Data quality is poor, and there is no simple way for trust validation.*” (P26); and “*We need skilled people who can work with these technologies, but right now we do not have enough.*” (P19). Requirements centered on building foundational and interoperable infrastructure, standardizing and integrating health information systems/EHR, improving data quality and validation, and capacity-building/continuing education: “*We must connect different information systems… interoperability between them can be extremely beneficial.*” (P25); “*A genuine reform of electronic health records is required.*” (P17); “We need noise-free and purified datasets.” (P32); and “*We urgently need to provide training on AI use for physicians, health professionals, and policymakers*” (P25).

- Service delivery

The dataset identified ineffectiveness of AI in remote areas and inequitable access to services as key delivery-level challenges. No discrete, delivery-specific requirements were articulated for this dimension; instead, participants framed enabling conditions under governance, financing, and resource generation (e.g., infrastructure, data integration, and payment reforms) as prerequisites for equitable AI-supported PHC. AI-enabled service delivery was thus perceived as an outcome of broader system readiness rather than an independent operational focus. As one participant emphasized, “*A national strategy must be developed for AI use, covering all aspects of this technology, to improve health services at various levels. This strategy should include clear goals, regulatory mechanisms, and continuous monitoring*” (P25). Another noted that “*reforming tariff systems, payment mechanisms, and incentive models is necessary to improve performance*” (P21). Together, these accounts indicate that participants viewed improvements in service delivery as contingent upon upstream governance, strategic planning, and financing reforms.

### 3.2. Comparative Analysis

**Figure 2:**
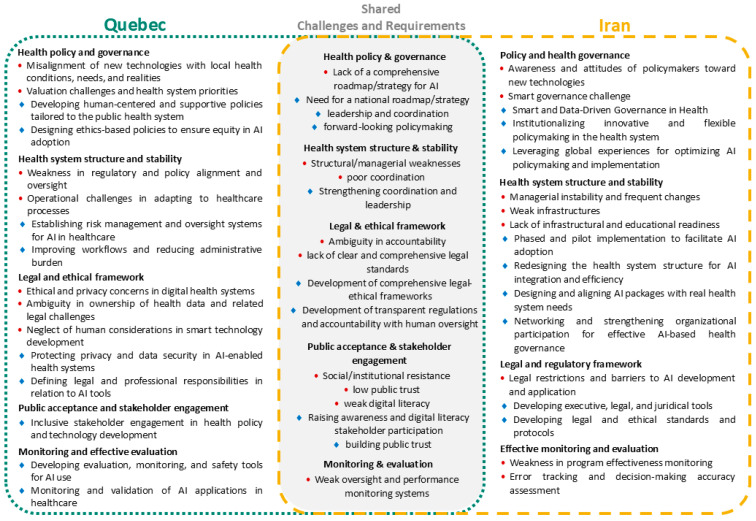
Comparative mapping of AI-related challenges and requirements in PHC under the stewardship dimension of the PCET framework, showing country-specific and shared themes identified in Quebec and Iran. Red circles indicate challenges, while blue diamond symbols represent requirements.

**Figure 3:**
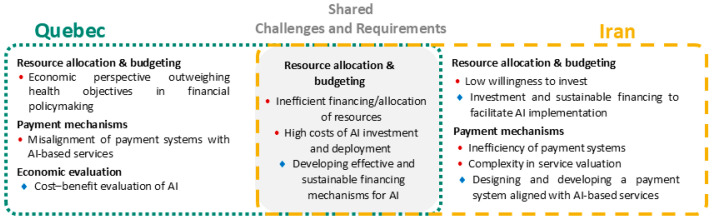
Comparative mapping of AI-related challenges and requirements in PHC under the financing & incentives dimension of the PCET framework, showing country-specific and shared themes identified in Quebec and Iran. Red circles indicate challenges, while blue diamond symbols represent requirements.

**Figure 4:**
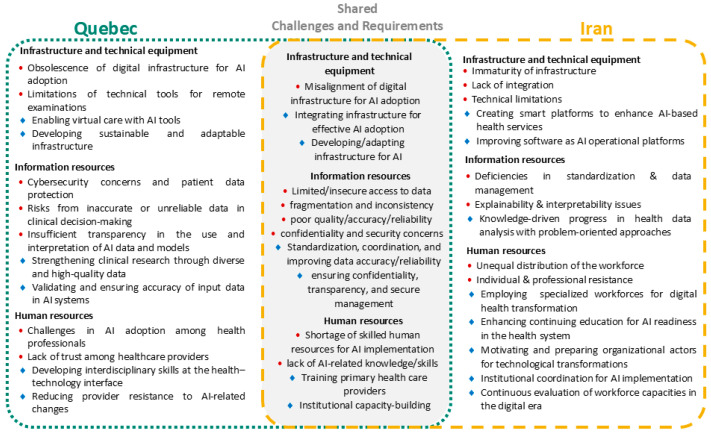
Comparative mapping of AI-related challenges and requirements in PHC under the resource generation dimension of the PCET framework, showing country-specific and shared themes identified in Quebec and Iran. Red circles indicate challenges, while blue diamond symbols represent requirements.

**Figure 5:**
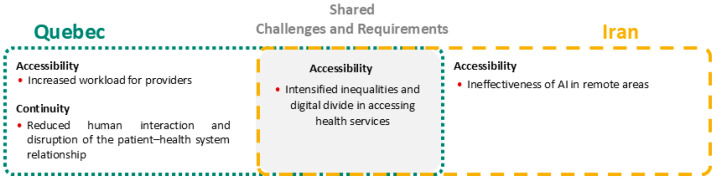
Comparative mapping of AI-related challenges and requirements in PHC under the service delivery dimension of the PCET framework, showing country-specific and shared themes identified in Quebec and Iran. Red circles indicate challenges, while blue diamond symbols represent requirements.

This section presents a cross-case comparative analysis of the findings from Quebec and Iran, structured according to the PCET framework. The analysis focuses on the four core dimensions, for each, a visual synthesis (Figure 2, Figure 3, Figure 4 and Figure 5) illustrates the challenges and requirements identified in both contexts, highlighting areas of overlap as well as country-specific differences. These figures provide a concise mapping of the comparative results, complementing the detailed country-specific findings reported in the previous section.

#### 3.2.1. Stewardship

As illustrated in Figure 2, both Quebec and Iran share several stewardship-related challenges, including the lack of a comprehensive roadmap for AI, structural and managerial weaknesses, unclear legal and accountability frameworks, limited public trust and digital literacy, and weak monitoring systems. Accordingly, both contexts highlight the need for national strategies, stronger leadership and coordination, comprehensive legal–ethical frameworks, stakeholder engagement, and robust evaluation mechanisms. In Quebec, specific concerns centered on the misalignment of technologies with local needs, administrative delays, operational barriers, and ethical and privacy issues, with requirements emphasizing human-centered and equity-oriented policies, risk management, and improved workflows. In Iran, distinctive challenges related to policymakers’ limited readiness, managerial instability, weak infrastructures, and restrictive laws, while requirements focused on smart and data-driven governance, phased pilot implementations, structural redesign, and the development of regulatory tools and standards. Overall, while convergence exists around strategy, governance, and accountability, Quebec reflects operational and ethical concerns, whereas Iran emphasizes governance reform and infrastructural preparedness.

#### 3.2.2. Financing & Incentives

Financing emerged as a critical constraint across both health systems, driven by high AI investment costs and inefficient financial structures, highlighting the shared need for sustainable and adaptive funding mechanisms. Figure 3 illustrates how these shared constraints manifest differently across contexts. Quebec’s context is characterized by the predominance of economic considerations over health objectives and misaligned payment systems, with cost–benefit evaluation emphasized as a requirement. In Iran, distinctive issues include low willingness to invest, inefficiency and complexity in payment systems, and the priority of designing financing channels aligned with AI-based services.

#### 3.2.3. Resource Generation

As depicted in Figure 4, resource generation shows the highest degree of overlap across the two contexts. Both face persistent infrastructures, data fragmentation, poor quality of health data, concerns over confidentiality and security, and shortages of skilled human resources for AI implementation. Accordingly, both contexts emphasized the need to strengthen infrastructures, improve data standardization and reliability, ensure transparency and secure data management, and build institutional capacity and training programs. In Quebec, context-specific barriers included cybersecurity risks, insufficient transparency in AI interpretation, and challenges in building trust among healthcare providers, with requirements highlighting virtual care with AI tools, sustainable infrastructures, and clinical research with diverse datasets. In Iran, distinct challenges were linked to deficiencies in standardization, explainability issues, unequal workforce distribution, and professional resistance, while requirements focused on developing smart platforms, improving operational software, employing specialized digital health workforces, and enhancing continuous education and organizational readiness. Overall, while both contexts converge on infrastructure, data, and human resource needs, Quebec places more weight on clinical transparency and provider acceptance, whereas Iran prioritizes structural modernization, explainability, and workforce transformation.

#### 3.2.4. Service Delivery

Service delivery indicates that both Quebec and Iran face significant equity-related challenges in ensuring fair access to AI-enabled health services, as illustrated in Figure 5. A shared concern is the risk of intensified inequalities and the persistence of the digital divide, which may marginalize vulnerable populations. In Quebec, the main issues are the increased workload for healthcare providers and the reduced human interaction that undermines the patient–provider relationship, raising questions about the human-centeredness of AI-driven care. By contrast, in Iran, the most pressing challenges are the ineffectiveness of AI tools in remote areas and the persistence of inequitable access to services, reflecting infrastructural and geographic disparities. Overall, while both contexts converge on the risk of widening inequalities, Quebec’s emphasis lies in provider burden and relational continuity, whereas Iran’s focus is on geographic inequities and the limited effectiveness of AI in underserved regions. These divergent service delivery concerns directly reflect the systemic readiness differences identified in preceding dimensions. In Quebec, challenges related to provider workload and relational continuity arise from the complexities of integrating AI into already mature clinical workflows, linking service delivery tensions to issues of stewardship and resource generation. In contrast, in Iran, concerns regarding geographic inequity and limited tool effectiveness stem from foundational gaps in digital infrastructure and resource generation, reinforcing that delivery-level challenges are downstream manifestations of broader system readiness.

## 4. Discussion

This study provides comparative, empirically grounded insights into the systemic and contextual determinants of AI implementation in PHC, across two distinct health systems, Quebec and Iran, using the PCET framework as an analytical lens. By juxtaposing a high-resource, digitally mature system with a middle-resource, developing system, the study expands current understanding of how governance capacity, financing mechanisms, infrastructural readiness, and service design interact to shape national trajectories toward AI-enabled PHC. The findings underscore that AI readiness is not merely a function of technological capability but rather the outcome of adaptive governance, sustainable financing, robust infrastructures, and workforce preparedness. These findings highlight that AI readiness in PHC unfolds through context-specific sequencing and trade-offs, reinforcing that effective AI integration depends not only on the presence of key capacities but on aligning their development trajectories with system maturity and institutional readiness. While previous research has encompassed global reviews as well as single-country studies examining specific AI applications in healthcare [49,50,51], this study fills an existing gap in comparative research on AI readiness in PHC, offering actionable insights for policymakers seeking context-sensitive pathways for digital transformation.

In interpreting these findings, attention should be paid to the composition of the study sample. Differences in participants’ professional roles across the two settings may have shaped how challenges related to AI integration in PHC were articulated. In Iran, the greater representation of managerial and administrative actors may have contributed to a greater emphasis on governance arrangements, financing mechanisms, and system-level coordination. In contrast, participants in Quebec, who were more frequently embedded in academic, technical, and innovation-oriented environments, more often highlighted issues related to technical readiness, data infrastructure, and regulatory safeguards. This contextual lens is important for interpreting the comparative patterns that follow. In addition, although AI was intentionally defined broadly in the interview guide to capture a wide range of experiences with digital and intelligent systems in PHC, AI applications vary substantially in maturity and clinical risk. Lower-risk applications, such as administrative automation or basic chatbots, primarily depend on organizational readiness and workflow integration, whereas more advanced applications, including predictive models and clinical decision support systems, impose higher demands related to data quality, regulatory oversight, and clinical accountability. The barriers identified in this study should therefore be interpreted as system-level readiness constraints that may manifest differently across this spectrum.

### 4.1. Systemic Patterns and Contextual Divergences

Across both systems, AI readiness emerged as a function of systemic coherence rather than isolated technological adoption. Shared constraints, fragmented strategies, inefficient financing mechanisms, uncoordinated data systems, and persistent digital inequities mirror global experiences documented in other health systems [52,53,54]. Convergent requirements also point to actionable reforms: developing coherent national strategies, strengthening cross-sectoral coordination, ensuring sustainable funding, and building interoperable infrastructures supported by skilled human resources.

However, contextual divergences are equally pronounced. Quebec faces operational and ethical challenges, particularly in aligning AI tools with clinical workflows, maintaining transparency, and fostering professional trust. Corresponding requirements emphasize human-centered policymaking, ethical oversight, and collaborative governance mechanisms. Iran’s barriers are more structural, rooted in managerial instability, weak legal frameworks, and immature digital infrastructures. Its associated requirements focus on establishing smart and data-driven governance structures, comprehensive legal frameworks, and foundational investment in infrastructure and workforce training. These contrasts reaffirm that while digital maturity defines the type of challenges, governance capacity determines the system’s ability to respond.

It should also be acknowledged that the PCET framework is not an AI-specific readiness model. Accordingly, and given the intentionally broad definition of AI adopted in this study, the analysis primarily captures systemic, digital, and organizational readiness conditions for AI integration in PHC, rather than readiness for the implementation of specific AI models or applications. This focus may partly explain the cross-cutting and system-level nature of the identified requirements and conclusions.

#### 4.1.1. Stewardship

Governance capacity emerged as the linchpin of successful AI implementation. Both settings suffer from fragmented oversight, inconsistent accountability, and limited public engagement. These findings align with global reports highlighting the critical role of strategic governance, ethical oversight, and stakeholder participation in ensuring trustworthy AI adoption [10,55,56].

In Quebec, the governance deficit is procedural rather than structural: overly rigid regulations and the lack of ethical harmonization impede agile adaptation. The prioritization of human-centered, equity-oriented policies echoes calls in the literature for “human-in-the-loop” governance approaches that preserve professional autonomy and patient safety [57,58]. Within this context, an underlying tension emerges between the pace of AI-driven innovation and the need for robust regulatory oversight and ethical harmonization. As AI applications are introduced into already mature clinical and organizational workflows, stewardship challenges increasingly revolve around balancing innovation momentum with safety, accountability, and professional trust. In contrast, Iran’s gap is structural: managerial instability, fragmented policymaking, and the absence of a coherent national AI policy hinder strategic continuity. Similar observations have been reported in other middle-resource contexts, such as India and Brazil, where national digital health strategies remain at early stages [21,59]. Therefore, Iran’s required reforms include establishing a National AI Council for Health, developing an integrated roadmap, and embedding ethical and legal frameworks tailored to the local sociotechnical context. Beyond earlier literature that framed stewardship as a supportive function, this study demonstrates it as a determinant shaping all subsequent readiness dimensions [60,61]. Governments should institutionalize anticipatory AI governance bodies that ensure ethical alignment, stakeholder participation, and policy coherence across the health system.

#### 4.1.2. Financing and Incentives

Economic readiness remains a critical bottleneck globally [62,63], and this study illustrates how its form varies by resource context. In Quebec, the predominance of economic logic over health value mirrors concerns in other high-income countries where cost–benefit frameworks often fail to capture long-term clinical and social gains from AI adoption [64]. Participants’ emphasis on robust cost–benefit evaluations aligns with international recommendations for evidence-based digital health investment strategies [65,66]. In Iran, limited investment capacity, outdated payment mechanisms, and weak financial incentives constrain the ability to mobilize resources. These findings echo prior studies showing that health systems with centralized financing and fragmented insurance structures face greater difficulty in sustaining digital transformation [66,67]. The call for hybrid financing models and incentive schemes tailored to underserved regions reflects a pragmatic awareness of how financing reform must accompany governance and infrastructural readiness. Within this context, a key underlying tension emerges between the need for upfront investment in digital infrastructure and AI capabilities and the reluctance of insurers and funding bodies to support such initiatives in the absence of clear governance frameworks and demonstrated returns. This tension constrains financing sequencing, as service-level AI deployment remains dependent on prior reforms in payment mechanisms, incentive structures, and public investment commitment.

Taken together, these results suggest that while Quebec grapples with optimizing investment efficiency, Iran’s priority lies in establishing foundational mechanisms to make digital health investment viable and equitable. Accordingly, health ministries should embed AI financing within long-term PHC budgets, align reimbursement with service outcomes, and integrate AI cost–benefit assessment into public investment planning.

#### 4.1.3. Resource Generation

This dimension exhibited the strongest cross-context convergence. In both settings, deficits in digital infrastructure, data quality, and human resource capacity directly limit absorptive capacity for AI. These findings reaffirm the global consensus that AI maturity depends not on isolated technological progress but on the interaction between reliable data systems and skilled professionals [52,68].

In Quebec, issues such as limited interoperability, cybersecurity vulnerabilities, and professional skepticism underscore the importance of trust-enabling infrastructures, resonating with studies that identify trust and transparency as decisive for clinical acceptance of AI [69,70]. Requirements such as ensuring reliable data governance, improving dataset representativeness, and promoting interdisciplinary training are aligned with prior studies [71,72]. In Iran, infrastructural immaturity and data fragmentation are more acute. Weak EHR integration, non-standardized coding systems, and a lack of AI-literate staff create dependency on external vendors, a pattern also documented in similar LMIC contexts [73,74]. Addressing these requires a coordinated strategy for EHR standardization, secure data-sharing protocols, and large-scale workforce development initiatives within medical universities and PHC networks. Beyond confirming existing findings, this study reveals the sequencing logic of capacity-building: technical, data, and human infrastructures form a reinforcing triad that underpins all other readiness domains. Countries should prioritize concurrent investment in interoperable infrastructure, data quality assurance, and interdisciplinary workforce training as the backbone of sustainable AI integration.

#### 4.1.4. Service Delivery

At the delivery level, both systems demonstrate how technological innovation can inadvertently amplify inequities if introduced without system readiness, consistent with the broader literature [75]. Participants in Quebec expressed concerns over workload intensification and diminished relational care, echoing global debates about digital burnout and depersonalization [76,77]. In Iran, challenges were primarily infrastructural and geographic, reflecting persistent inequities in access to technology and connectivity, particularly in rural and underserved regions. The absence of explicit delivery-level requirements in both contexts indicates that equity-oriented AI integration remains a downstream function of broader systemic readiness, suggesting that advances in service equity are contingent on prior reforms in governance, financing, and resource generation. This sequential dependency supports arguments that service-level benefits of AI in PHC are emergent outcomes of system-level readiness, rather than isolated technological interventions [20,78]. Policymakers should embed equity monitoring tools in all AI deployments and pursue phased, context-sensitive implementation strategies that match infrastructural and human capacities.

Synthesizing across PCET dimensions, the comparative findings highlight that AI implementation in PHC follows a sequential and cumulative trajectory. Governance reform establishes legitimacy and coordination; financing mechanisms support sustainability; resource generation provides operational capacity; and service delivery reflects the visible impact of systemic readiness. Quebec’s pathway is characterized by refining integration and ethical oversight within a mature digital ecosystem, whereas Iran’s pathway emphasizes foundational system-building and governance modernization. Together, they illustrate that digital transformation is not a universal blueprint, but a context-dependent progression shaped by institutional learning and resource asymmetry. Cross-national collaboration, through shared data governance standards, international training programs, and bilateral pilot projects, can promote reciprocal learning and narrow the AI readiness gap between high- and middle-resource systems.

Building on this synthesis, the findings reveal a set of underlying tensions that shape AI implementation and require active navigation by policymakers. In Quebec, a central tension exists between the pressure for rapid innovation and the presence of deliberately slow administrative and regulatory processes designed to ensure patient safety, ethical legitimacy, and professional accountability. This tension underscores the importance of adaptive regulatory approaches, such as iterative approval pathways and regulatory sandboxes, that can balance innovation with oversight. A related paradox emerges between the aspiration for human-centered AI and concerns about increased clinical workload, pointing to the need for workflow co-design and workforce support mechanisms to ensure that AI-enabled tools alleviate rather than intensify operational pressures.

In Iran, the analysis points to structural tensions rooted in political economy and system capacity. While large-scale investment in digital infrastructure and AI capability is widely recognized as essential, insurers’ limited willingness to invest constrains system-wide implementation, highlighting the need for financing models that better align incentives across public payers, providers, and technology developers. This challenge is compounded by tensions between centralized governance ambitions and operational instability, suggesting the value of phased implementation strategies and pilot-based scaling to build institutional capacity while managing risk. Collectively, these dialectics underscore that AI readiness is not merely a technical challenge, but a governance process shaped by trade-offs between speed and safety, ambition and feasibility, and short-term constraints and long-term system transformation.

From a methodological perspective, data were collected through written responses rather than live interviews. While this approach facilitated participation among time-constrained experts, it may have limited opportunities for real-time probing and clarification, potentially affecting the depth and interactivity of the data. At the same time, written responses allowed participants to provide more deliberate and structured reflections. This trade-off should be considered when interpreting the findings, particularly with respect to nuanced experiential aspects of AI integration in PHC.

### 4.2. Conceptual and Global Implications

This study contributes to global literature by empirically linking PCET domains to stages of AI readiness and revealing how national contexts shape implementation trajectories. As shown in prior research, organizational readiness is a complex, multifaceted challenge that must be carefully considered for successful AI implementation in healthcare [79], this analysis conceptualizes it as an evolving policy ecosystem influenced by governance, economics, and social trust. Quebec exemplifies the tension between innovation and ethical legitimacy, while Iran highlights the foundational reforms required to achieve digital equity.

Beyond these two cases, the findings underscore that governance maturity, not technological availability, determines sustainable AI adoption in PHC. Cross-national partnerships that facilitate knowledge co-production can take concrete forms, such as joint pilot projects between PHC organizations, shared regulatory learning platforms, and co-developed implementation guidelines tailored to PHC settings. Similarly, global data collaboratives in the PHC context may involve federated data-sharing models, common data standards for PHC records, and cross-country benchmarking initiatives that enable learning without compromising data sovereignty or patient privacy. Regional AI training networks that link academic institutions, PHC providers, and policymakers can further support capacity-building by aligning workforce competencies with real-world service delivery needs. Together, these mechanisms can accelerate progress toward equitable AI-enabled PHC while respecting contextual differences. Future strategies should therefore frame AI not as a disruptive tool but as a catalyst for institutional learning, enabling adaptive, inclusive, and resilient PHC systems.

### 4.3. Limitations and Future Directions

This study has several limitations. First, structural and contextual differences between Quebec’s and Iran’s PHC systems limit direct comparability, as institutional designs, financing models, and digital maturity differ substantially, and technological ecosystems are shaped by distinct contextual conditions. Second, in the Iranian context, the technological ecosystem is influenced by external constraints, including international sanctions, limited access to global cloud computing services and advanced hardware, and dependence on foreign AI and large language model providers. These factors constrain the feasibility, design choices, and pace of AI implementation in PHC and represent a significant contextual difference from Quebec that should be considered when interpreting findings related to infrastructural and technical barriers. Third, the qualitative nature of the study introduces potential selection bias due to purposive sampling. In the Iranian context, this included an exclusively male sample, reflecting prevailing gender patterns in senior managerial and policy-making roles. This may limit the transferability of the findings, particularly regarding frontline service delivery perspectives, which are largely represented by female health workers such as Behvarzes. In addition, although a shared and broad definition of AI was used in the interview guides, participants may have interpreted the concept differently based on their professional background and exposure to digital technologies. This variability reflects the exploratory nature of the study and may have influenced how challenges and requirements were articulated across contexts. In addition, differences in participant profiles across the two settings may have influenced the comparative findings. The greater representation of technical and academic stakeholders in Quebec and managerial or administrative actors in Iran suggests that some identified “barriers” may reflect differences in respondent perspectives and professional vantage points, rather than purely system-level differences. This should be considered when interpreting the comparative patterns. Finally, given the rapid evolution of AI policies and technologies, some findings may evolve over time.

Despite these limitations, the study’s comparative design enhances conceptual transferability by offering a framework that other countries can adapt to assess AI readiness in PHC. Future research should pursue mixed-method and longitudinal approaches to trace how AI integration evolves within PHC systems over time. Additionally, the development of standardized AI readiness benchmarks encompassing governance, financing, infrastructure, and workforce indicators could facilitate international comparison and policy alignment.

## 5. Conclusions

This comparative analysis highlights that successful AI implementation in PHC depends less on the specific technologies adopted and more on how they are integrated within existing health system structures. Applying the PCET framework to Quebec and Iran, and drawing directly on empirical qualitative patterns observed across stewardship, financing, resource generation, and service delivery domains, revealed that governance capacity, sustainable financing, robust infrastructure, and skilled human resources collectively determine readiness trajectories. High-resource systems must refine ethical alignment and operational integration, while middle-resource settings require foundational reforms and workforce development, as reflected in the contrasting empirical challenges and requirements identified across the two cases. Rather than a universal model, AI integration should follow context-specific and phased strategies that align with institutional maturity.

Policymakers should embed AI within national PHC strategies, ensure stable financing, and foster cross-national learning to promote equity and trust, drawing on the system-level barriers and enablers identified in this study. Beyond these cases, the PCET framework can serve as a practical diagnostic tool, enabling countries to systematically assess their AI readiness across key system dimensions and to identify context-appropriate pathways for implementation. Ultimately, sustainable digital transformation demands an anticipatory and inclusive approach that balances innovation with ethics and social responsibility.

## Figures and Tables

**Table 1:** Inclusion and exclusion criteria for the selection of expert participants in Quebec and Iran, adapted to reflect differences in PHC system structures and digital health roles.

Country	Inclusion Criteria	Exclusion Criteria
Quebec	≥18 years old At least 1 year of experience in the PHC system (clinical, academic, or managerial role) Family physicians, faculty members, or experts in digital health/AI with relevance to PHC Active interest in PHC and emerging technologies Ability to participate in English or French Willingness to provide informed consent	No relevant experience in PHC or AI Exclusively hospital-based professionals without PHC linkage Students or trainees lacking practical experience Inability or unwillingness to provide consent
Iran	≥18 years old At least 1 year of experience in the PHC system (clinical, academic, or managerial role) Health managers, family physicians, community health workers (behvarzes), faculty members, or AI experts with relevance to PHC Publications, committee membership, or recognized expertise in PHC/AI (for academic participants) Ability to participate in Persian Willingness to provide informed consent	No relevant experience in PHC or AI Exclusively hospital-based professionals without PHC linkage Students or trainees lacking practical experience Inability or unwillingness to provide consent

**Table 2:** Demographic and professional characteristics of participants in Quebec and Iran.

Quebec	Iran
Gender Male: 10 (66.7%) Female: 5 (33.3%)	Gender Male: 16 (100%) Female: 0 (0%)
Age 25–44 years: 5 (33.3%) 45–64 years: 10 (66.7%)	Age 25–44 years: 3 (18.7%) 45–64 years: 13 (81.3%)
Educational level Bachelor: 1 (6.6%) PhD: 11 (73.3%) Postdoc: 3 (20%)	Educational level PhD: 15 (93.7%) Postdoc: 1 (6.2%)
Field of expertise Computer Science and AI: 1 (6.66%) Public Health: 2 (13.3%) Neuroscience: 3 (20%) Digital Health: 4 (26.6%) Biomedical Engineering: 1 (6.66%) Nutrition Science: 2 (13.3%) Family medicine: 2 (13.3%)	Field of expertise PHC ^1^: 7 (43.7%) AI ^2^: 1 (6.25) Health Foresight: 4 (25%) Family Medicine: 1 (6.25%) Health Policy & Management: 2 (15.5%) Health IT: 1 (6.25%)
Organizational role Faculty member: 8 (53.3%) Senior researcher: 2 (13.3%) Research assistant 3 (20%) Physician: 1 (6.66%) AI specialist: 1 (6.66%)	Organizational role Faculty member: 9 (53.89%) Organization director: 4 (25%) Deputy director: 2 (12.5%) Technology officer: 1 (6.25%)
Work experience <5 years: 4 (26.7%) 6–10 years: 1 (6.7%) 11–15 years: 5 (33.3%) >16 years: 5 (33.3%)	Work experience 11–15 years: 1 (6.2%) >16 years: 15 (93.7%)
Institutions Laval University: 12 (80%) McGill University: 1 (6.66%) Mila Institute: 1 (6.66%) CIUSSSCN: 1 (6.66%)	Institutions Tehran UMS ^3^: 3 (18.75%) Kerman UMS: 2 (12.5%) Kerman Health Insurance: 5 (31.25%) MHME ^4^ (PHC Deputy): 2 (12.5%) Tehran University: 2 (12.5%) Isfahan UMS: 1 (6.25%) Iranian Futures Studies Association: 1 (6.25%)
Type of participation Online interviews: 8 (53.3%) Written interviews: 7 (46.6%)	Type of participation Online interviews: 7 (43.7%) Written interviews: 2 (12.5%) Focus group: 7 (43.7%)

^1^ PHC = Primary Health Care; ^2^ AI = Artificial Intelligence; ^3^ UMS = University of Medical Sciences; ^4^ MHME = Ministry of Health and Medical Education. (Percentages may not total 100% due to rounding).

**Table 3:** Challenges and requirements for AI implementation in PHC in Quebec, structured by the dimensions of the PCET framework.

**PCET** **Dimension**	• **Challenges** **Main Theme** • Sub-Theme	♦ **Requirements** **Main Theme** ♦ Sub-Theme
**Stewardship**	**Health policy and governance** • Misalignment of new technologies with local health conditions, needs, and realities • Lack of a comprehensive roadmap for AI implementation in the health system • Valuation challenges and health system priorities	**Health policy and governance** ♦ Developing human-centered and supportive policies tailored to the public health system ♦ Designing ethics-based policies to ensure equity in AI adoption ♦ Establishing a coordinated strategy for national and global AI governance
	**Health system structure and stability** • Slow administrative procedures for AI deployment in the health system • Weakness in regulatory and policy alignment and oversight • Structural resistance to adopting new technologies • Operational challenges in adapting to healthcare processes	**Health system structure and stability** ♦ Establishing risk management and oversight systems for AI in healthcare ♦ Strengthening leadership, coordination, and national collaboration for AI deployment in health ♦ Improving workflows and reducing administrative burden
	**Legal and ethical framework** • Ethical and privacy concerns in digital health systems • Lack of clear and comprehensive legal and regulatory frameworks • Ambiguity in accountability for AI-based decisions • Ambiguity in ownership of health data and related legal challenges • Neglect of human considerations in smart technology development	**Legal and ethical framework** ♦ Protecting privacy and data security in AI-enabled health systems ♦ Developing transparent regulations and oversight systems for AI control and evaluation ♦ Defining legal and professional responsibilities in relation to AI tools ♦ Integrating ethical and human-centered principles in AI development and application
	**Collaboration and interaction among stakeholders** • Lack of participation and collaboration among stakeholders • Cultural and perceptual challenges in public acceptance of AI • Low public trust in technological tools and companies • Failure in public information and digital literacy promotion	**Public acceptance and stakeholder engagement** ♦ Cross-sectoral and interdisciplinary collaboration among stakeholders ♦ Inclusive stakeholder engagement in health policy and technology development ♦ Public education and awareness for AI acceptance ♦ Enhancing transparency, public trust, and social participation in digital health governance
	**Monitoring and effective evaluation** • Weaknesses in frameworks for AI evaluation and effectiveness	**Monitoring and effective evaluation** ♦ Developing evaluation, monitoring, and safety tools for AI use ♦ Monitoring and validation of AI applications in healthcare
**Financing & Incentives**	**Resource allocation and budgeting** • Inefficiency in the financing structure of AI projects • High costs of developing and deploying AI technologies • Economic perspective outweighing health objectives in financial policymaking **Payment mechanisms** • Misalignment of payment systems with AI-based services	**Resource allocation & budgeting** ♦ Developing effective financing mechanisms for AI innovation **Economic evaluation** ♦ Cost–benefit evaluation of AI
**Resource Generation**	**Infrastructure and technical equipment** • Obsolescence and misalignment of digital infrastructure for AI adoption • Limitations of technical tools for remote examinations **Information resources** • Cybersecurity concerns and patient data protection • Limited and insecure access to data • Fragmentation and inconsistency of health data at different levels • Risks from inaccurate or unreliable data in clinical decision-making • Insufficient transparency in the use and interpretation of AI data and models **Human resources** • Shortage of skilled human resources for AI implementation • Challenges in AI adoption among health professionals • Lack of awareness, trust, and insufficient training among healthcare providers	**Infrastructure and technical equipment** ♦ Integrating infrastructure for effective AI adoption ♦ Enabling virtual care with AI tools ♦ Developing sustainable and adaptable infrastructure **Information resources** ♦ Ensuring confidentiality, transparency, and user consent in health data use ♦ Secure and transparent data management in digital health systems ♦ Strengthening clinical research through diverse and high-quality data ♦ Validating and ensuring accuracy of input data in AI systems **Human resources** ♦ Training and capacity-building of primary care providers in AI health applications ♦ Developing interdisciplinary skills at the health–technology interface ♦ Reducing provider resistance to AI-related changes
**Service Delivery**	**Accessibility** • Increased workload for providers • Intensified inequalities and digital divide in accessing health services **Continuity** • Reduced human interaction and disruption of the patient–health system relationship	

Legends: Circle sign: Challenges; Diamond sign: Requirements.

**Table 4:** Challenges and requirements for AI implementation in PHC in Iran, structured by the dimensions of the PCET framework.

**PCET** **Dimension**	• **Challenges** **Main Theme** • Sub-Theme	♦ **Requirements** **Main Theme** ♦ Sub-Theme
**Stewardship**	**Policy and health governance** • Awareness and attitudes of policymakers toward new technologies • National strategy and strategic planning • Smart governance challenges **Health system structure and stability ** • Managerial instability and frequent changes • Weak managerial structures and infrastructures • Weak national and international coordination • Lack of infrastructural and educational readiness **Legal and regulatory framework** • Responsibility and accountability of AI • Legal restrictions and barriers to AI development and application • Incompatibility of laws and lack of legal standards **Public acceptance and stakeholder engagement** • Institutional and social resistance to technology adoption • Lack of public awareness and digital literacy • Public trust and social concerns • Social participation and negative social implications **Effective monitoring and evaluation** • Weaknesses in oversight and performance evaluation • Weakness in program effectiveness monitoring • Error tracking and decision-making accuracy assessment	**Policy and health governance** ♦ Developing a national roadmap and strategy for AI implementation ♦ Smart and Data-Driven Governance in Health ♦ Facilitating targeted and forward-looking policymaking for AI ♦ Strengthening leadership, commitment, and national participation for AI implementation ♦ Institutionalizing innovative and flexible policymaking in the health system ♦ Leveraging global experiences for optimizing AI policymaking and implementation
		**Health system structure and stability** ♦ Phased and pilot implementation to facilitate AI adoption ♦ Redesigning the health system structure for AI integration and efficiency ♦ Designing and aligning AI packages with real health system needs ♦ Strengthening intersectoral collaboration for synergistic AI implementation ♦ Networking and strengthening organizational participation for effective AI-based health governance ♦ Expanding international interactions for AI development
		**Legal and regulatory framework** ♦ Developing comprehensive legal and ethical frameworks for AI use ♦ Developing executive, legal, and juridical tools ♦ Developing legal and ethical standards and protocols ♦ Enhancing standardization and human oversight in AI implementation
		**Public acceptance and stakeholder engagement** ♦ Raising awareness, public education, and cultural readiness for AI adoption ♦ Strengthening social capital and public trust in technological innovation ♦ Networking and strengthening interdisciplinary expert participation in AI implementation
**Financing & Incentives**	**Resource allocation & budgeting** • Inefficient allocation of resources • High investment costs • Low willingness to invest **Payment mechanisms** • Inefficiency of payment systems • Complexity in service valuation	**Resource allocation & budgeting** ♦ Investment and sustainable financing to facilitate AI implementation **Payment mechanisms** ♦ Designing and developing a payment system aligned with AI-based services
**Resource Generation**	**Infrastructure & technical equipment** • Immaturity of infrastructure • Lack of coordination & integration • Technical limitations **Information resources** • Lack of integration & coordination of data • Deficiencies in standardization & data management • Poor quality, accuracy & reliability of health data • Restricted access to data • Security & confidentiality concerns • Explainability & interpretability issues **Human resources** • Unequal distribution of the workforce • Lack of specialized workforce • Lack of AI-related knowledge & skills • Individual & professional resistance	**Infrastructure and technical equipment** ♦ Developing basic infrastructure to support AI implementation ♦ Creating smart platforms to enhance AI-based health services ♦ Improving software as AI operational platforms **Information resources** ♦ Improving the quality, accuracy, and reliability of data for AI analysis ♦ Standardization, coordination, and integration of health information systems ♦ Knowledge-driven progress in health data analysis with problem-oriented approaches **Human resources** ♦ Training and employing a specialized workforce for digital health transformation ♦ Enhancing continuing education for AI readiness in the health system ♦ Motivating and preparing organizational actors for technological transformations ♦ Institutional capacity-building and coordination for AI implementation ♦ Continuous evaluation of workforce capacities in the digital era
**Service Delivery**	**Accessibility** • Ineffectiveness of AI in remote areas • Inequitable access to services	

Legends: Circle sign: Challenges; Diamond sign: Requirements.

## Data Availability

The qualitative datasets generated and analyzed during the current study are not publicly available due to ethical and confidentiality restrictions, but are available from the corresponding author on reasonable request.

## Supplementary Materials

The following supporting information can be downloaded at: https://www.mdpi.com/article/10.3390/healthcare14020145/s1.

## Abbreviations

The following abbreviations are used in this manuscript:

AI	Artificial Intelligence
PHC	Primary Health Care
EHR	Electronic Health Record
PCET	Primary Care Evaluation Tool
FMGs	Groupes de Médecine de Famille
UMS	University of Medical Sciences
MHME	Ministry of Health and Medical Education

## References

[1] Maleki Varnosfaderani S., Forouzanfar M. (2024). The role of AI in hospitals and clinics: Transforming healthcare in the 21st century. Bioengineering.

[2] Silcox C., Zimlichmann E., Huber K., Rowen N., Saunders R., McClellan M., Kahn C.N., Salzberg C.A., Bates D.W. (2024). The potential for artificial intelligence to transform healthcare: Perspectives from international health leaders. npj Digit. Med..

[3] Kumar A., Gupta A., Raj U. New Era of Intelligent Medicine: Future Scope and Challenges. Proceedings of the 2024 11th International Conference on Reliability, Infocom Technologies and Optimization (Trends and Future Directions) (ICRITO).

[4] Kalra N., Verma P., Verma S. (2024). Advancements in AI based healthcare techniques with FOCUS ON diagnostic techniques. Comput. Biol. Med..

[5] Patel D., Kore S.A. (2020). Artificial intelligence: Future impacts, challenges and recommendations on healthcare services. Int. J. Community Med. Public Health.

[6] Jiang F., Jiang Y., Zhi H., Dong Y., Li H., Ma S., Wang Y., Dong Q., Shen H., Wang Y. (2017). Artificial intelligence in healthcare: Past, present and future. Stroke Vasc. Neurol..

[7] Nivethitha V., Daniel R., Surya B., Logeswari G. (2025). Empowering public health: Leveraging AI for early detection, treatment, and disease prevention in communities—A scoping review. J. Postgrad. Med..

[8] Yousefi F., Naye F., Ouellet S., Yameogo A.-R., Sasseville M., Bergeron F., Ozkan M., Cousineau M., Amil S., Rhéaume C. (2025). Artificial Intelligence in Health Promotion and Disease Reduction: Rapid Review. J. Med. Internet Res..

[9] Leon N., Xu H. (2023). Implementation considerations for non-communicable disease-related integration in primary health care: A rapid review of qualitative evidence. BMC Health Serv. Res..

[10] Yousefi F., Dehnavieh R., Laberge M., Gagnon M.-P., Ghaemi M.M., Nadali M., Azizi N. (2025). Opportunities, challenges, and requirements for Artificial Intelligence (AI) implementation in Primary Health Care (PHC): A systematic review. BMC Prim. Care.

[11] Sasseville M., Yousefi F., Ouellet S., Naye F., Stefan T., Carnovale V., Bergeron F., Ling L., Gheorghiu B., Hagens S. (2025). The Impact of AI Scribes on Streamlining Clinical Documentation: A Systematic Review. Healthcare.

[12] Qin H., Tong Y. (2025). Opportunities and challenges for large language models in primary health care. J. Prim. Care Community Health.

[13] Alami H., Lehoux P., Papoutsi C., Shaw S.E., Fleet R., Fortin J.-P. (2024). Understanding the integration of artificial intelligence in healthcare organisations and systems through the NASSS framework: A qualitative study in a leading Canadian academic centre. BMC Health Serv. Res..

[14] Sachar S., Dakessian M., Beitari S., Badrinarayanan S. (2020). Artificial intelligence alongside physicians in Canada: Reality and risks. J. Sci. Policy Gov..

[15] Ribeiro L., O’Brien T. (2025). Data-driven decision-making: Turning insights into action. J. Clin. Urol..

[16] Shirjang A., Doshmangir L., Assan A. (2020). Exploring Current Iran’s Primary Healthcare System: Challenges and Solutions. https://api.semanticscholar.org/CorpusID:240648768.

[17] Mehrolhassani M.H., Yazdi-Feyzabadi V., Dehnavieh R., Bahaadinbeigy K., Kargar M. (2025). Barriers to Telemedicine Establishment in Iran: A Systematic Review. Iran. J. Public Health.

[18] Shirjang A., Mahfoozpour S., Asl I.M., Doshmangir L. (2020). Challenges and strategies of implementation rural family physician in iran: A qualitative study. Depiction Health.

[19] Shojaee-Mend H., Mahi M., Khajavi A., Maleki M.S., Nabiolahi A. (2024). The potential use of digital health in Iran: A systematic mapping review. Front. Health Inform..

[20] Dehnavieh R., Inayatullah S., Yousefi F., Nadali M. (2025). Artificial Intelligence (AI) and the future of Iran’s Primary Health Care (PHC) system. BMC Prim. Care.

[21] Pillai A. (2023). Artificial Intelligence in Healthcare Systems of low-and Middle-Income countries: Requirements, gaps, challenges, and potential strategies. Int. J. Appl. Health Care Anal..

[22] Zhao R.C., Yuan X. AI in Healthcare for Resource Limited Settings: An Exploration and Ethical Evaluation. Proceedings of the Companion Proceedings of the ACM on Web Conference.

[23] Lapointe J., Badr J., Motulsky A. (2021). Usage of Digital Tools to Access Primary Care in Quebec: An Environmental Scan. Public Health and Informatics.

[24] Tabrizi J.S., Pourasghar F., Nikjoo R.G. (2017). Status of Iran’s primary health care system in terms of health systems control knobs: A review article. Iran. J. Public Health.

[25] Tavassoli M. (2008). Iranian health houses open the door to primary care. Bull. World Health Organ..

[26] Takian A., Doshmangir L., Rashidian A. (2013). Implementing family physician programme in rural Iran: Exploring the role of an existing primary health care network. Fam. Pract..

[27] Kueper J.K., Terry A.L., Zwarenstein M., Lizotte D.J. (2020). Artificial intelligence and primary care research: A scoping review. Ann. Fam. Med..

[28] Rahimi S., Archambault P., Zomahoun H.T.V., Chandavong S., Gagnon M.-P., Wong S., Sharma G., Langlois L., Rheault N., Couturier Y. (2022). Application of AI in community based primary health care: Systematic review and critical appraisal. Ann. Fam. Med..

[29] Creswell J.W., Poth C.N. (2016). Qualitative Inquiry and Research Design: Choosing Among Five Approaches.

[30] Stake R.E. (1995). The Art of Case Study Research.

[31] Greenhalgh T., Wherton J., Papoutsi C., Lynch J., Hughes G., Hinder S., Fahy N., Procter R., Shaw S. (2017). Beyond adoption: A new framework for theorizing and evaluating nonadoption, abandonment, and challenges to the scale-up, spread, and sustainability of health and care technologies. J. Med. Internet Res..

[32] Denzin N.K., Lincoln Y.S. (2011). The Sage Handbook of Qualitative Research.

[33] Tong A., Sainsbury P., Craig J. (2007). Consolidated criteria for reporting qualitative research (COREQ): A 32-item checklist for interviews and focus groups. Int. J. Qual. Health Care.

[34] Breton M., Levesque J.-F., Pineault R., Hogg W. (2011). L’implantation du modèle des groupes de médecine de famille au Québec: Potentiel et limites pour l’accroissement de la performance des soins de santé primaires. Prat. Et Organ. Des Soins.

[35] Wankah P., Breton M., Lukey A., Gaboury I., Smithman M.A., Malo M.-É., Oelke N. (2022). Shaping Primary Health Care Teams and Integrated Care in Québec: An Overview of Policies (2000–2020). Health Reform. Obs.–Obs. Réformes Santé.

[36] Alami H., Lehoux P., Attieh R., Fortin J.-P., Fleet R., Niang M., Offredo K., Rouquet R., Ag Ahmed M.A., Ly B.A. (2021). A “not so quiet” revolution: Systemic benefits and challenges of telehealth in the context of COVID-19 in Quebec (Canada). Front. Digit. Health.

[37] Kueper J.K., Pandit J.A. (2025). Artificial Intelligence for Healthcare in Canada: Contrasting Advances and Challenges. HealthcarePapers.

[38] Marier Tetrault E., Aver B Ribeiro P., El Haffaf M., Bechard S., Brouillard P., Bebawi E., Yuan S., Hanh Vo T.X., Khazaka M., Nguyen J. (2023). The Impact of Remote Patient Monitoring and Digital Therapeutics on Major Clinical Events and Costs in Heart Failure Patients: Early Experience in the Quebec Public Healthcare System. Circulation.

[39] Marier-Tétrault E., Bebawi E., Béchard S., Brouillard P., Zuchinali P., Remillard E., Carrier Z., Jean-Charles L., Nguyen J.N.K., Lehoux P. (2024). Remote patient monitoring and digital therapeutics enhancing the continuum of care in heart failure: Nonrandomized pilot study. JMIR Form. Res..

[40] Yazdi-Feyzabadi V., Delavari S., Ghasemi S. (2018). Primary care in Iran needs a paradigm shift. Br. J. Gen. Pract..

[41] Shadpour K. (2000). Primary health care networks in the Islamic Republic of Iran. East. Mediterr. Health J..

[42] Maghsoudloo M., Abolhassani F., Lotfibakhshaiesh N. (2016). Connecting primary health care: A comprehensive pilot study. Acta Medica Iran..

[43] Starfield B., Shi L., Macinko J. (2005). Contribution of primary care to health systems and health. Milbank Q..

[44] Braun V., Clarke V. (2006). Using thematic analysis in psychology. Qual. Res. Psychol..

[45] Lincoln Y.S., Guba E.G. (1985). Naturalistic Inquiry.

[46] (2010). Primary Care Evaluation Tool. https://publichealth.jhu.edu/johns-hopkins-primary-care-policy-center/primary-care-assessment-tools.

[47] Miles M.B., Huberman A.M. (1994). Qualitative Data Analysis: An expanded Sourcebook.

[48] Huberman A.M. (1994). An Expanded Sourcebook Qualitative Data Analysis. https://vivauniversity.wordpress.com/wp-content/uploads/2013/11/milesandhuberman1994.pdf.

[49] Kellermann A.L., Jones S.S. (2013). What it will take to achieve the as-yet-unfulfilled promises of health information technology. Health Aff..

[50] Nasr S.S., Gouda N.A., Khater A.I., Mostafa O.A. (2025). Knowledge, Perception, Practice and Barriers to Use Artificial Intelligence (AI) among Egyptian Medical Doctors. Egypt. J. Nutr. Health.

[51] Ciecierski-Holmes T., Singh R., Axt M., Brenner S., Barteit S. (2022). Artificial intelligence for strengthening healthcare systems in low-and middle-income countries: A systematic scoping review. npj Digit. Med..

[52] Castonguay A., Wagner G., Motulsky A., Paré G. (2024). AI maturity in health care: An overview of 10 OECD countries. Health Policy.

[53] Yi S., Yam E.L.Y., Cheruvettolil K., Linos E., Gupta A., Palaniappan L., Rajeshuni N., Vaska K.G., Schulman K., Eggleston K.N. (2024). Perspectives of digital health innovations in low-and middle-income health care systems from South and Southeast Asia. J. Med. Internet Res..

[54] Ahmed M.M., Okesanya O.J., Olaleke N.O., Adigun O.A., Adebayo U.O., Oso T.A., Eshun G., Lucero-Prisno D.E. (2025). Integrating Digital Health Innovations to Achieve Universal Health Coverage: Promoting Health Outcomes and Quality Through Global Public Health Equity. Healthcare.

[55] Madanchian M., Taherdoost H. (2025). Ethical theories, governance models, and strategic frameworks for responsible AI adoption and organizational success. Front. Artif. Intell..

[56] Goktas P., Grzybowski A. (2025). Shaping the future of healthcare: Ethical clinical challenges and pathways to trustworthy AI. J. Clin. Med..

[57] Gilbert S., Anderson S., Daumer M., Li P., Melvin T., Williams R. (2023). Learning from experience and finding the right balance in the governance of artificial intelligence and digital health technologies. J. Med. Internet Res..

[58] Rozenblit L., Price A., Solomonides A., Joseph A.L., Srivastava G., Labkoff S., Debronkart D., Singh R., Dattani K., Lopez-Gonzalez M. (2025). Towards a multi-stakeholder process for developing responsible AI governance in consumer health. Int. J. Med. Inform..

[59] Filgueiras F., Junquilho T.A. (2023). The Brazilian (Non) perspective on national strategy for artificial intelligence. Discov. Artif. Intell..

[60] Guan J. (2019). Artificial intelligence in healthcare and medicine: Promises, ethical challenges and governance. Chin. Med. Sci. J..

[61] Gati R.A., Rizki M., Posumah R.Y. (2021). Readiness of Artificial Intelligence to Accelerate Bureaucratic Reform in Indonesia. MAP Obs. J. Penelit. Adm. Publik.

[62] El Arab R.A., Abdulaziz O., Sagbakken M. (2025). Economic, ethical, and regulatory dimensions of artificial intelligence in healthcare: An integrative review. Front. Public Health.

[63] McCoy L.G., Bihorac A., Celi L.A., Elmore M., Kewalramani D., Kwaga T., Martinez-Martin N., Prôa R., Schamroth J., Shaffer J.D. (2025). Building health systems capable of leveraging AI: Applying Paul Farmer’s 5S framework for equitable global health. BMC Glob. Public Health.

[64] Hendrix N., Veenstra D.L., Cheng M., Anderson N.C., Verguet S. (2022). Assessing the economic value of clinical artificial intelligence: Challenges and opportunities. Value Health.

[65] Alami H., Lehoux P., Auclair Y., de Guise M., Gagnon M.-P., Shaw J., Roy D., Fleet R., Ag Ahmed M.A., Fortin J.-P. (2020). Artificial intelligence and health technology assessment: Anticipating a new level of complexity. J. Med. Internet Res..

[66] Nguyen K.-H., Comans T., Nguyen T.T., Simpson D., Woods L., Wright C., Green D., McNeil K., Sullivan C. (2024). Cashing in: Cost-benefit analysis framework for digital hospitals. BMC Health Serv. Res..

[67] Barsky N.P. (2021). Successful Digital Transformation Hinges on 5 C-Suite Questions. IT Profr..

[68] Gazquez-Garcia J., Sánchez-Bocanegra C.L., Sevillano J.L. (2025). AI in the Health Sector: Systematic Review of Key Skills for Future Health Professionals. JMIR Med. Educ..

[69] Ranwala R., Andrade A.Q. (2025). Enhancing AI Clinical Decision Support Trust: Design Workshop Insights from General Practitioners. Stud. Health Technol. Inform..

[70] Shevtsova D., Ahmed A., Boot I.W., Sanges C., Hudecek M., Jacobs J.J., Hort S., Vrijhoef H.J. (2024). Trust in and acceptance of artificial intelligence applications in medicine: Mixed methods study. JMIR Hum. Factors.

[71] Paprica P.A., Crichlow M., Maillet D.C., Kesselring S., Pow C., Scarnecchia T.P., Schull M.J., Cartagena R.G., Cumyn A., Dostmohammad S. (2023). Essential requirements for the governance and management of data trusts, data repositories, and other data collaborations. Int. J. Popul. Data Sci..

[72] Janssen M., Brous P., Estevez E., Barbosa L.S., Janowski T. (2020). Data governance: Organizing data for trustworthy Artificial Intelligence. Gov. Inf. Q..

[73] Mwogosi A., Mambile C. (2025). AI integration in EHR systems in developing countries: A systematic literature review using the TCCM framework. Inf. Discov. Deliv..

[74] Kumar M., Mostafa J. (2019). Research evidence on strategies enabling integration of electronic health records in the health care systems of low-and middle-income countries: A literature review. Int. J. Health Plan. Manag..

[75] Sovacool B.K., Newell P., Carley S., Fanzo J. (2022). Equity, technological innovation and sustainable behaviour in a low-carbon future. Nat. Hum. Behav..

[76] Wroclawski M., Heldwein F.L. (2021). Editorial comment: Digital physician burnout in the “new normal” workplace. J. Endourol..

[77] Hessari H., Bai A., Daneshmandi F. (2024). Generative AI: Boosting adaptability and reducing workplace overload. J. Comput. Inf. Syst..

[78] Katonai G., Arvai N., Mesko B. (2025). AI and primary care: Scoping review. J. Med. Internet Res..

[79] Alami H., Lehoux P., Denis J.-L., Motulsky A., Petitgand C., Savoldelli M., Rouquet R., Gagnon M.-P., Roy D., Fortin J.-P. (2021). Organizational readiness for artificial intelligence in health care: Insights for decision-making and practice. J. Health Organ. Manag..

