# Supplementary material for: Integrating Artificial Intelligence (AI) in Primary Health Care (PHC) Systems: A Framework-Guided Comparative Qualitative Study
Source: Healthcare (Basel). 2026 Jan 7;14(2):145. doi: 10.3390/healthcare14020145 (PMC12840649; doi:10.3390/healthcare14020145)
Supplement: Supplementary file 1 [file healthcare-14-00145-s001.zip › healthcare-4030416-supplementary/Supplementary Material S1-COREQ Checklist.pdf]

COREQ (COnsolidated criteria for REporting Qualitative research) Checklist

A checklist of items that should be included in reports of qualitative research. You must report the page number in your manuscript where you consider each of the items listed in this checklist. If you have not included this information, either revise your manuscript accordingly before submitting or note N/A.

Topic	Item No.	Guide Questions/Description	Reported on Page No.
**Domain 1: Research team and reflexivity**			
Personal characteristics			
Interviewer/facilitator	1	Which author/s conducted the interview or focus group?	Methods – Data collection/ p 8
Credentials	2	What were the researcher's credentials? E.g. PhD, MD	Methods – Data collection/ p 8
Occupation	3	What was their occupation at the time of the study?	Not reported
Gender	4	Was the researcher male or female?	Not reported
Experience and training	5	What experience or training did the researcher have?	Not reported
Relationship with participants			
Relationship established	6	Was a relationship established prior to study commencement?	Methods – Participants / p 4
Participant knowledge of the interviewer	7	What did the participants know about the researcher? e.g. personal goals, reasons for doing the research	Methods – Ethics / P 7
Interviewer characteristics	8	What characteristics were reported about the inter viewer/facilitator? e.g. Bias, assumptions, reasons and interests in the research topic	Method- Credibility and trustworthiness of the data/ P 9
**Domain 2: Study design**			
Theoretical framework			
Methodological orientation and Theory	9	What methodological orientation was stated to underpin the study? e.g. grounded theory, discourse analysis, ethnography, phenomenology, content analysis	Methods – Study design/ P 3 and 4
Participant selection			
Sampling	10	How were participants selected? e.g. purposive, convenience, consecutive, snowball	Method- Participants / P 4
Method of approach	11	How were participants approached? e.g. face-to-face, telephone, mail, email	Method- Data collection/ P 7
Sample size	12	How many participants were in the study?	Method- Participants and Table 2/ 5 and 6
Non-participation	13	How many people refused to participate or dropped out? Reasons?	Not reported
Setting			
Setting of data collection	14	Where was the data collected? e.g. home, clinic, workplace	Method- setting – P 4
Presence of nonparticipants	15	Was anyone else present besides the participants and researchers?	Not reported

Description of sample	16	What are the important characteristics of the sample? e.g. demographic data, date	Method- Participants and Table 2/ 5 and 6
Data collection			
Interview guide	17	Were questions, prompts, guides provided by the authors? Was it pilot tested?	Method- Data collection; Supplementary Material S2/ P 8
Repeat interviews	18	Were repeat interviews carried out? If yes, how many?	No repeat interviews
Audio/visual recording	19	Did the research use audio or visual recording to collect the data?	Methods – Data collection / P 8
Field notes	20	Were field notes made during and/or after the interview or focus group?	Methods – Data collection/ P 8
Duration	21	What was the duration of the interviews or focus group?	Method- Methods – Data collection/ P 8
Data saturation	22	Was data saturation discussed?	Method- Methods – Data collection/ P 8
Transcripts returned	23	Were transcripts returned to participants for comment and/or	Not reported
**Topic**	**Item No.**	**Guide Questions/Description**	**Reported on Page No.**
		correction?	
**Domain 3: analysis and findings**			
Data analysis			
Number of data coders	24	How many data coders coded the data?	Methods – Data analysis and Results- P 9 and 13
Description of the coding tree	25	Did authors provide a description of the coding tree?	Results- P 9 to 19
Derivation of themes	26	Were themes identified in advance or derived from the data?	Results- Table 3 and 4
Software	27	What software, if applicable, was used to manage the data?	Method-data analysis/ P 9
Participant checking	28	Did participants provide feedback on the findings?	Not reported
Reporting			
Quotations presented	29	Were participant quotations presented to illustrate the themes/findings?<br>Was each quotation identified? e.g. participant number	Results – Thematic findings/ P 10, 13, and 14
Data and findings consistent	30	Was there consistency between the data presented and the findings?	Results – Comparative findings / P 17-19 and Discussion – Integration of findings / P 19-24
Clarity of major themes	31	Were major themes clearly presented in the findings?	Results- Table 3 and 4
Clarity of minor themes	32	Is there a description of diverse cases or discussion of minor themes?	Results- Table 3 and 4

Developed from: Tong A, Sainsbury P, Craig J. Consolidated criteria for reporting qualitative research (COREQ): a 32-item checklist for interviews and focus groups. *International Journal for Quality in Health Care*. 2007. Volume 19, Number 6: pp. 349 – 357
