# Supplementary material for: Integrating Artificial Intelligence (AI) in Primary Health Care (PHC) Systems: A Framework-Guided Comparative Qualitative Study
Source: Healthcare (Basel). 2026 Jan 7;14(2):145. doi: 10.3390/healthcare14020145 (PMC12840649; doi:10.3390/healthcare14020145)

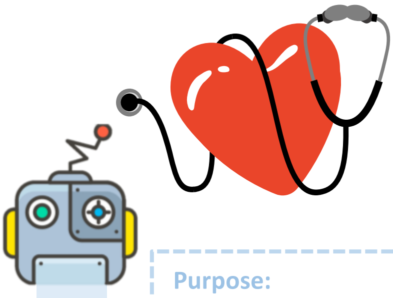

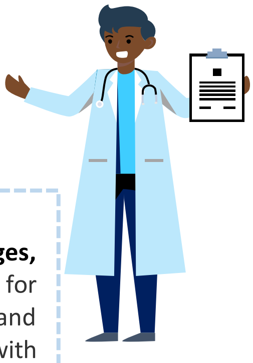

Purpose:

This study, **"Artificial Intelligence (AI) Implementation in Primary Care (PC) Setting: Challenges, Opportunities, and Requirements",** aims to identify challenges, requirements, and suggestions for implementing AI in PC systems, which can help the health system to be aware and prepare in facing and implementing AI- based technologies and applications. In this regard, this interview in 4 sections and with 10 open-ended questions is provided to ask your valuable and helpful experience in reaching to the aim of the study.

Before Starting to Answer: **Please make sure you complete the Consent Form**

Researcher Information:

- Supervisors: ***Prof. Maude Laberge; Prof. Reza Dehnavieh,***
- Principal Investigator: ***Farzaneh Yousefi***
- Position: ***Research Intern***
- Contact Information: farzaneh.yousefi.1@ulaval.ca **, 514 802 4854**
- Affiliation: *Ph.D. Candidate in Health Services Management**,** Research Intern,* Department of Social and Preventive Medicine, Faculty of Medicine, Laval University, Quebec City, Quebec, Canada.

- A translator will help the researcher translate the participants' speech from French to English or Persian.

- One phase of this study has been funded by Mitacs.
- This project has been approved by the Research Ethics Board of Université Laval: Approval No. 2023-443/29-02-2024

Thanks for Your Collaboration

Biographical Questions:

Name: **Gender:** Female Male Prefer not to say

Age: 18 to 24 years 25 to 44 years 45 to 64 years 65 years and over

Level of Education: **Field of Education:**

Work Position/ Expertise: **Years of Work Experiences/ Expertise:**

Name of Organization: **Region:**

Introduction Question

- Can you provide an overview of your experience and involvement in the Canada/Quebec's Primary Care (PC) system, especially in researching or working with AI-based technologies or applications?

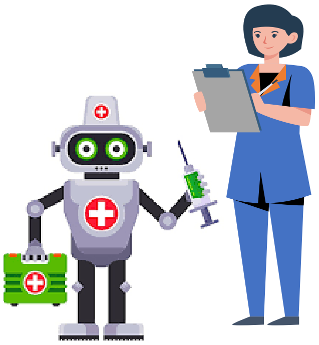

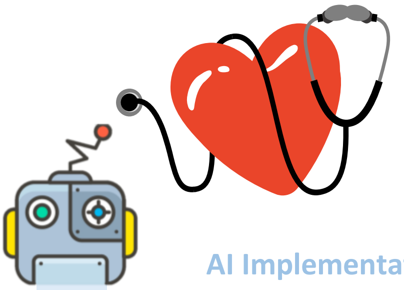

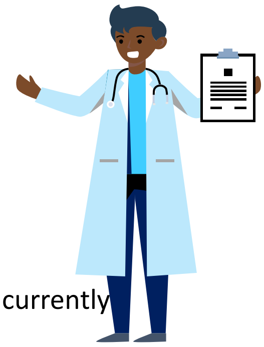

AI Implementation in PHC

Successful AI Integration

- Could you share any specific examples of successful AI-based technologies or applications currently used in Canada/Quebec's PC system? Is there any training or guidelines for using these?

- What are the reasons for using these technologies routinely in the context of Primary Care?

Impact and Benefits

- In your experience, what measurable benefits or impacts have AI-based solutions brought to primary care in Canada/Quebec's?

- How do the collaborations between academia, industry, and healthcare institutions foster the development and deployment of AI-based solutions?

Challenges and Requirements

- What are the primary challenges or barriers faced when implementing AI in the PC system in Canada/Quebec's?

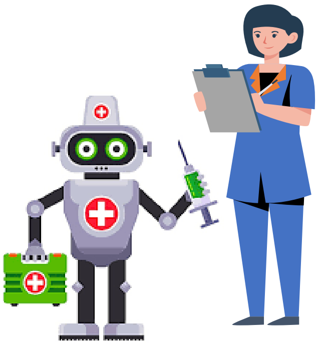

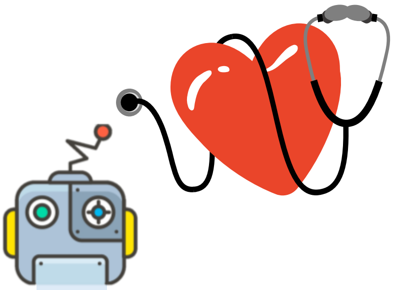

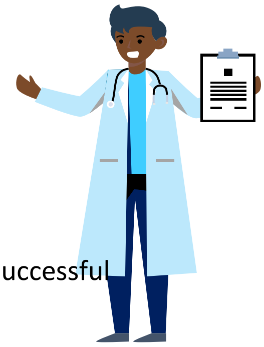

- Are there specific requirements or conditions that need to be in place for the successful implementation of AI in PC setting?

Suggestions and Future Outlook

- Are there specific areas where AI could further enhance or revolutionize primary care setting?

- Are there any considerations that should be focused on in Implementing AI in primary Care?

- What kind of policies or support from the government or healthcare institutions would facilitate the effective implementation of AI in Primary Care?

Thanks for Your Collaboration

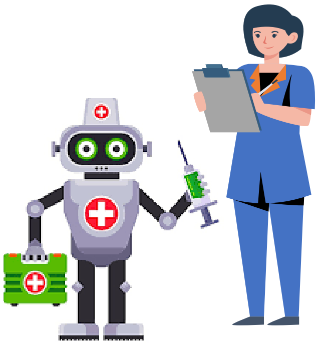

Supplement: Supplementary file 1 [file healthcare-14-00145-s001.zip › healthcare-4030416-supplementary/Supplementary Material S2-Interview Questions.pdf]
