# Supplementary material for: Integrating Artificial Intelligence (AI) in Primary Health Care (PHC) Systems: A Framework-Guided Comparative Qualitative Study
Source: Healthcare (Basel). 2026 Jan 7;14(2):145. doi: 10.3390/healthcare14020145 (PMC12840649; doi:10.3390/healthcare14020145)
Supplement: Supplementary file 1 [file healthcare-14-00145-s001.zip › healthcare-4030416-supplementary/Supplementary Material S3-Tables.pdf]

Challenges of AI implementation in Quebec's PHC

Table S1. Challenges of AI implementation in Quebec's PHC

PCET Dimension	Main Theme	Sub-Theme	Codes	Quote
Stewardship	Health policy and governance	Misalignment of new technologies with local health conditions, needs, and realities	Digital solutions not aligned with patient needs	"Developing digital solutions is time-consuming, and by the time implementation occurs, their features may no longer meet the current needs of users." (P2)
			Alignment with healthcare professionals' needs	"There must be alignment between the implemented tools and the initial needs of professionals (listening to their needs by technology developers)." (P3)
			Complexity of healthcare environments	"The complexity of healthcare environments." (P6)
			Health challenges beyond technology	"Health challenges often lie beyond technology." (P7)
			Complexity of local issues	"The complexity of local issues." (P8)
			Misalignment between AI tools and me*islands* dical needs	"Even if an application is excellent, if it is unusable for physicians, it will not be adopted." (P10)
		Lack of a comprehensive roadmap for AI implementation in the health system	Time-consuming development of digital solutions	"The development of digital solutions is time-consuming. In citizen- and patient-partnered projects, collaborators may drop out, requiring us to restart the recruitment and integration process." (P2)
			No perceived necessity for AI implementation	"There is no real necessity for implementing AI systems." (P7)
			Absence of a comprehensive strategy for AI integration	"We need a clear strategy for AI in primary care… Currently, innovations are like small islands with no overarching vision." (P11)
			Lack of mechanisms for large-scale AI implementation	"Most AI projects remain at a small scale and lack the capacity for widespread deployment." (P11)
		Valuation challenges and health system priorities	Hospitals becoming economic organizations rather than focusing on patient health	"The risk of the current system is that it operates like a business… and we lose the primary goal, which is people's health." (P14)
	Health system structure and stability	Slow administrative procedures for AI deployment in the health system	Industrial partner withdrawal	"An industrial partner may decide to withdraw at any stage of development." (P2)
			Lengthy approval process for AI tools in Canada	"In Germany, three months of data is required for approval… In Canada, this process takes two years." (P10)
			Slow bureaucratic processes for AI integration in Canada's health system	"At the pace we are going, it will take 10 years before this tool is truly available to anyone." (P13)
		Weakness in regulatory and policy alignment and oversight	Constantly changing regulatory frameworks	"Regulatory frameworks for AI in health are constantly evolving." (P4)
			Lack of institutional collaboration	"There is no joint collaboration between health institutions." (P6)
			Lack of regulatory frameworks for managing continuously learning AI tools	"The major difference now is that AI tools can relearn after deployment… but there are still no guidelines for this." (P13)

		Structural resistance to adopting new technologies	Resistance of the pharmaceutical industries to adopting AI	"Most of the time, AI belongs to the domain of open science… but pharmaceutical companies have not yet fully entered this field." (P10)
			Resistance of pharmaceutical companies to adopting open science	"Pharmaceutical companies have not yet fully engaged with open science." (P10)
			Resistance of pharmaceutical companies to open collaborations	"Even if an application is excellent, if it is unusable for physicians, it will not be adopted." (P10)
			Resistance of medical organizations and professional associations	"There are strong vested interests in the medical field… and persuading them to accept change is difficult." (P13)
			Resistance to change and adopting new technologies in healthcare systems	"Healthcare providers may resist adopting new technologies if they do not understand their benefits or are not involved in their development." (P15)
		Operational challenges in adapting to healthcare processes	Challenges of AI integration	"Integrating AI into existing systems faces challenges of interoperability and compatibility." (P4)
			Lack of preparedness among healthcare providers	"Not all healthcare providers are trained to work with AI or understand its applications." (P11)
			Complexity in aligning AI with PHC processes	"AI tools often disrupt patient care pathways and require changes in care processes." (P11)
			Changes in physicians' work methods	"Changes in physicians' work methods." (P1)
			Limitations in performing physical examinations	"I do not have sufficient tools to support physical examination." (P12)
	Legal and ethical framework	Ethical and privacy concerns in digital health systems	Ethical concerns	"Ethical concerns regarding the integration of AI into health." (P4)
			Concerns about data ownership	"There are issues related to transparency… all of these need to be improved in the private sector." (P9)
			Concerns about the misuse of data by insurers or pharmaceutical companies	"What happens if my data ends up on the dark web? … It could be sold to a pharmaceutical company… but I also see potential benefits for research." (P9)
			Concerns about protecting patient privacy in new systems	"I accidentally shared a patient's name in an email, which raised concerns about privacy protection." (P12)
			Concerns about emotional recognition algorithms and patients' physiological data	"If advanced systems can analyze micro-expressions of emotions… people should be aware that machines can read this information." (P14)
			Concerns about over-reliance on AI recommendations	"Recommendations provided by AI may not always be appropriate from the perspective of health professionals managing patients." (P15)
			Risk of excessive reliance on digital data without in-person examinations	"If health professionals rely only on digital data and do not see the patient in person, this could lead to misdiagnosis or inappropriate treatments." (P15)
		Lack of clear and comprehensive legal and regulatory frameworks	Lack of a specific legal framework for AI tools approval and integration	"The regulatory framework for AI in medicine." (P13)
			Complexity of legal processes for AI medical devices	"Getting approval for a device is relatively simple… but integrating it into the public health system is very difficult." (P13)

			Security challenges and patient privacy concerns in digital systems	“Hackers can hack the system and disclose patient information, which is a security concern.” (P15)
		Ambiguity in accountability for AI-based decisions	Lack of transparency in AI-related decision-making processes	“We need to explain to people what we are going to do and why… in order to build trust.” (P10)
			Ethical and legal pressures in AI-based decision-making	“AI should support decision-making, but the final responsibility rests with health professionals.” (P11)
			Ethical and privacy concerns in using facial and emotion recognition algorithms	“If our emotions have certain markers… people must know that machines can read them in order to accept and interact with this technology.” (P14)
			Uncertainty regarding identity in virtual consultations	“In video or telephone consultations, it is sometimes difficult to ensure that the person we are speaking with is the actual patient.” (P15)
		Ambiguity in ownership of health data and related legal challenges	Intellectual property and open data challenges	“AI belongs to the domain of open science… but pharmaceutical companies have not yet fully entered this area.” (P10)
			Ethical and legal issues related to patient data access	“One of the ethical and social problems is access to data. But if there is an agreement and people give consent, this problem can be resolved.” (P13)
		Neglect of human considerations in smart technology development	Neglect of human issues in the early stages of AI development	“Those who think about humans and their emotions usually get involved too late… they should be engaged from day one of designing this technology.” (P14)
	Public acceptance and stakeholder engagement	Lack of participation and collaboration among stakeholders	Lack of collaboration with patients and citizens	“Project partners do not always know how to work with citizen and patient collaborators.” (P2)
			Lack of coordination between AI specialists and physicians	“Physicians know how to diagnose and prescribe… but they are not aware of AI, so we need to work together.” (P10)
			Collaboration challenges between GPs and other health professionals	“There may be disagreements between general practitioners and nurses, and sometimes they do not agree on how things should be done.” (P15)
			Coordination challenges between GPs and other professionals in adopting new technologies	“There may be challenges in collaboration between GPs, nurses, or other professionals in implementing new technologies.” (P15)
			Lack of interdisciplinary collaboration	“Interdisciplinary collaboration.” (P1)
		Cultural and perceptual challenges in public acceptance of AI	Social acceptance of new tools	“Social acceptance of new tools and the changes they bring.” (P3)
			Public fear of sharing health data	“Why are people so afraid of it… no one has been able to tell me what the real risk is.” (P9)
			Public resistance due to previous experiences (e.g., COVID vaccination)	“Despite a lot of information provided… part of the population was very afraid.” (P9)
			Public resistance to AI due to fear of the unknown	“When it comes to our health, we imagine risks like giant monsters… even though these risks are not clearly defined.” (P9)
			Public resistance to AI due to misunderstandings	“We need to explain to people what we want to do… so they do not think our goal is surveillance.” (P10)

			Public resistance and misunderstanding about AI's role	"AI is a tool to gather more information, not a replacement for healthcare providers." (P11)
			Cultural barriers and resistance in the healthcare system	"In the medical field, there are strong vested interests… and moving them to accept changes is difficult." (P13)
			Differences in AI adoption between Canada and the US	"The same tool is approved and integrated into the US system… but in Québec hospitals, it is not integrated." (P13)
		Low public trust in technological tools and companies	Low trust in private industries	"I trust universities more than the private sector… when profit is involved, there is always more risk of mistakes." (P9)
			Patients' trust issues with avatars and emotion-simulating machines	"Will people trust machines that simulate emotions? I am not sure, because people usually see emotions as contrary to rationality." (P14)
			Patients' trust issues with machines and AI systems	"Will people trust machines that simulate emotions?… People usually see emotions as contrary to rationality." (P14)
			Low patient trust in AI tools in medical consultations	"Patients may feel uncomfortable with AI being used in consultations, and they have the right to choose the type of care they receive." (P15)
			Risk of patient misguidance due to misuse of AI	"Patients need education to know how to use AI tools effectively without becoming overly dependent on them." (P11)
			Risks of AI chatbots in mental health	"There are documented cases where AI chatbots have promoted suicide, which is a serious issue." (P11)
		Failure in public information and digital literacy promotion	Lack of mechanisms for public information and trust-building	"People are afraid of sharing data because they do not understand its benefits and only see vague risks." (P9)
			Challenges in patient education and awareness about new AI tools	"If patients do not know what these tools do… professionals will also not move toward using them." (P14)
	Monitoring and effective evaluation	Weaknesses in frameworks for AI evaluation and effectiveness	Tools for measuring AI impact	"There are tools to measure the impact of AI on the quality of care and services." (P4)
			Lack of clear standards for AI evaluation	"There is no fixed framework for evaluating and approving AI tools in the health system." (P11)
			Difficulties in approving and integrating AI tools into public health systems	"In Canada, implementing AI tools in the public health system is very difficult." (P13)
Financing & Incentives	Resource allocation and budgeting	Inefficiency in the financing structure of AI projects	Financial challenges in securing funding	"Financing seems to me to be one of the main challenges." (P1)
			Lack of budget	"The budget may run out before the end of the project, forcing the team to stop until new funding is obtained." (P2)
			Lengthy funding and evaluation processes for AI projects	"The research cycle is very long… we need faster funding processes to conduct feasibility of new concepts." (P11)

		High costs of developing and deploying AI technologies	Lengthy and costly approval process for AI tools	"Getting approval for a relatively simple device is straightforward… but the main challenge for a company is entering the public health system." (P13)
			High costs of development, testing, and approval of AI tools in medicine	"Obtaining approval for a medical device from Health Canada costs between 3 to 5 million dollars… and even after that, no sales have yet been made." (P13)
		Economic perspective outweighing health objectives in financial policymaking	Excessive focus on economic savings instead of improving health	"The risk is that this technology will be seen only as a tool for saving time and resources… rather than truly improving the system." (P14)
			Problems linked to an economic approach in AI development in healthcare	"The risk is that hospitals operate like businesses… and instead of focusing on people's health, they only talk about costs and efficiency." (P14)
	Payment mechanisms	Misalignment of payment systems with AI-based services	Reimbursement code issues in Canada's health system for AI-based services	"A code for reimbursement must be created, which takes years." (P13)
Resource Generation	Infrastructure and Technical Equipment	Obsolescence and misalignment of digital infrastructure for AI adoption	Obsolescence of IT systems	"Obsolescence of IT systems." (P6)
			Challenges of adapting AI to health infrastructures	"The system is not adapted to technologies that are truly useful for patients." (P10)
			Incompatibility of health infrastructures with AI tools	"The system is not adapted to technologies that are truly useful for patients." (P10)
		Limitations of technical tools for remote examinations	Limitations of virtual physical examination tools	"I do not have sufficient tools, such as a camera to look into the ear, to support physical examinations during virtual consultations." (P12)
			Complexity of managing multiple medical platforms	"Using multiple platforms such as WhatsApp, Zoom, and separate calendars for managing tasks made work more difficult." (P12)
			Incompatibility of patient devices with physician systems	"The patient's device was not compatible with my EMR system, which required manual data integration." (P12)
			Incompatibility of patient tools and data with electronic systems	"Your device is not compatible with my EMR system." (P12)
			Difficulty integrating AI tools into public health systems in Canada	"Devices are approved in Canada, but they are not integrated into hospitals across Québec." (P13)
			Lack of interoperability among different electronic systems	"The absence of interoperability between different EMR systems makes data management more difficult for physicians." (P12)
	Information resources	Cybersecurity concerns and patient data protection	Respect for confidentiality and data consent	"The main challenge in using AI is respecting consent and confidentiality of personal data." (P2)
			Issues related to access, confidentiality, and data security	"Issues of access, confidentiality, and data security for building tools." (P4)
			Cybersecurity barriers and data protection	"Cybersecurity… at the moment is an issue that is blocking the whole progress." (P10)
			Risks of unintended disclosure through advanced algorithms	"If our emotions… have markers that reveal them… machines can read that without people knowing." (P14)

			Risks of unintended disclosure of patients' personal information due to physiological data analysis	"People must know that machines can read their emotions and physiological changes… otherwise, they will not participate in these interactions." (P14)
			Concerns about data security and the risk of hacking patient medical information	"There is a risk that hackers could access patient data stored in AI systems, raising concerns about privacy and security." (P15)
			Challenges related to patient data security in AI systems	"There are concerns that hackers could hack the system and access patient information." (P15)
		Limited and insecure access to data	Challenges of data access	"The main challenge is access to data." (P5)
			Difficulty accessing patients' personal data	"For an ordinary citizen, accessing their personal data is complex." (P9)
			Lack of access to adequate data for training algorithms	"How can we access data to train the algorithm?" (P10)
			Absence of proper structures for secure data access	"There is still no clear process for physicians to access data and develop projects." (P10)
			Privacy and ethical issues limiting access to medical data	"One of the ethical and social problems is access to data. But if there is an agreement and people consent, this problem can be solved." (P13)
			Lack of proper structure for secure access to health data	"I work in the health system… there is no place to securely share this data." (P9)
		Fragmentation and inconsistency of health data at different levels	Lack of integration in international health data management	"There is no secure place to share this data… we still depend on external tools like Google Drive for collaboration." (P9)
			Lack of integrated processes for data sharing	"There is still no clear process for physicians to access data and develop projects." (P10)
			Poor quality and fragmentation of health data	"Data are located in different places, and data quality is also problematic." (P11)
			Complexity of integrating diverse data into AI tools	"Combining different metrics is not mathematically simple and is complex." (P13)
			Lack of integration in hospital information systems and use of outdated methods	"Hospitals still use fax. It is a common joke, but it reflects a deeper reality… people still prefer paper boards because digital systems are not well designed." (P13)
		Risks from inaccurate or unreliable data in clinical decision-making	Accuracy and quality of input data in health monitoring apps	"If the information provided by the patient is not accurate, the system may produce an incorrect result that is not suitable for the patient." (P15)
			Need to ensure the accuracy of input data in AI tools	"If the input information from patients is incorrect, the AI system may give a wrong output that could be harmful for the patient." (P15)
			Uncertainty about patient identity in virtual consultations	"In video or telephone consultations, it is sometimes difficult to verify the identity of the actual patient." (P15)
			Uncertainty about the accuracy of patient input and its risks for clinical decisions	"If patient information is inaccurate, it can lead to wrong decisions, especially when relying on remote monitoring." (P15)
		Insufficient transparency in the use and interpretation of AI data and models	Issues of the AI black box	"Because of the black box that physicians cannot fully explain… this prevents them from engaging." (P9)
			Complexity in explaining the benefits and functioning of AI	"People need to understand what they are consenting to… but most regulations and processes are overly complex." (P9)

			Ambiguity about the use of data in research and associated risks	“What happens if my data ends up on the dark web?… but no one has been able to tell me what the real risk is.” (P9)
			Risk of misinterpretation of AI functioning	“There are barriers related to the perceptions of patients, physicians, or decision-makers, but these barriers are rapidly changing.” (P5)
	Human resources	Shortage of skilled human resources for AI implementation	Lack of qualified staff	“Lack of qualified staff (PHQ).” (P6)
			Shortage of staff to follow up on high-risk patients	“If we identify high-risk populations, do we have the capacity to follow them up?” (P11)
			Shortage of specialized staff for AI projects	“Innovation offices do not have enough resources and trained human resources to scale up projects.” (P11)
		Challenges in AI adoption among health professionals	Resistance of physicians to adopting new technologies	“My colleagues resist telemedicine, mainly because of the lack of physical examination.” (P12)
			Resistance of health professionals to change and new technologies	“Health professionals may resist adopting new tools if they do not trust the technology or were not involved in its development.” (P15)
			Physicians’ resistance to AI due to a lack of awareness	“Physicians cannot explain it well… this prevents them from being engaged in the process.” (P9)
			Slow and difficult acceptance of new technologies by healthcare professionals	“Healthcare professionals will not quickly change their ways of working unless they already know and believe in the technology.” (P14)
			Lack of widespread adoption of AI tools in Canadian hospitals	“Tools that are approved in the United States are integrated into their system… but in Québec, these tools are not integrated.” (P13)
			Non-acceptance of AI tools by healthcare professionals due to a lack of awareness and trust	“Healthcare professionals will not quickly change their ways of working unless they already know or believe in the technology.” (P14)
		Lack of awareness, trust, and insufficient training among healthcare providers	Need for training for health professionals	“Health professionals need training to be able to use the developed technological tools properly.” (P2)
			Limitations in skills and competencies	“There are limitations in individuals’ skills and competencies to appropriately use AI-based tools.” (P4)
			Lack of AI training for physicians and specialists	“Such training is still not offered in regular medical (MD) programs.” (P10)
			Challenges due to insufficient familiarity of health professionals with AI technologies	“Health professionals need training to understand how AI tools work, because lack of familiarity can be a barrier to adoption.” (P15)
			Non-acceptance of AI tools by healthcare professionals due to a lack of awareness and trust	“Healthcare professionals will not quickly change their ways of working unless they already know or believe in the technology.” (P14)
			Physicians’ resistance to AI due to a lack of awareness	“Physicians cannot explain it well… this prevents them from being engaged in the process.”(P9)
Service Delivery	Accessibility	Increased workload for providers	Administrative burdens	“Administrative burdens.” (P8)

		Intensified inequalities and digital divide in accessing health services	New inequalities due to limited access to AI	“AI tools are not suitable for everyone; not everyone feels comfortable using them.” (P11)
			Discrimination in access for patients with different conditions	“If patients do not know what these tools do and cannot request to use them… professionals will not adopt them either.” (P14)
			Risk of digital divides in access to health technologies for vulnerable populations	“Some communities may not have equal access to AI tools, leading to inequalities in health services.” (P15)
			Unequal access to AI tools for vulnerable or remote populations	“There may be inequalities in access to AI tools, especially for vulnerable populations or people living in remote areas.” (P15)
	Continuity	Reduced human interaction and disruption of the patient–health system relationship	Risk of reduced human interactions in care processes due to digital tools	“The risk is that this technology will be seen only as a tool for saving time and resources.” (P14)
			Risk of increased distance between healthcare systems and patients due to excessive digitalization	“Will people trust machines that simulate emotions? I am not sure… non-human interfaces raise many questions.” (P14)

Requirements of AI implementation in Quebec's PHC

Table S2. Requirements of AI implementation in Quebec's PHC

PCET	Main Them	Sub Theme	Codes	Quet
Stewardship	Health policy and governance	Developing human-centered and supportive policies tailored to the public health system	Adaptable technological tools	"Ensuring the development of a technological tool that can be adapted to meet the new needs of end-users." (P2)
			Responding to the initial needs of institutions	"Ensuring that the initial needs of institutions are addressed before proposing a technological solution." (P3)
			Technology is not a "magic solution"	"Technology is not a 'magic solution' to the problems of the health system." (P3)
			AI as a supportive tool, not a replacement	"AI is a tool that facilitates the management of care and services. It serves humans and cannot replace them. Let us humanize care and services." (P4)
			Patient-centered decision-making	"The decision should not be economic, but related to health or patient-centered… it must be considered from the patient's perspective." (P14)
			Patient-centered policy decisions for AI adoption	"Policy decisions on AI adoption should focus on improving patients' experiences and the quality of care, not only economic considerations." (P15)
			Patient-centered policy decisions for AI adoption	"Policy decisions on AI adoption must center on patients' experiences and needs, not merely economic or organizational considerations." (P15)
			Need for supportive policies to encourage AI use in healthcare	"Policymakers must ensure that AI is implemented to improve health outcomes, not solely for economic reasons." (P14)
		Designing ethics-based policies to ensure equity in AI adoption	Access, equity, and social justice	"Access, equity, social justice, and care." (P7)
			Balancing financial profit and public benefit in private research	"The private sector… needs greater balance in regulation and transparency." (P9)
			Policymakers' accountability to public concerns on AI	"Policymakers must respond to public questions… in order to change the system based on patients' needs." (P10)
		Establishing a coordinated strategy for national and global AI governance	Alignment with the goals of a global public health system	"And alignment with the goals of a global public health system." (P7)
			Improving AI governance	"I think adding indicators directly at the board level would be the most important and effective action." (P8)
			Developing a global AI strategy with regional adaptation	"We need a global plan for AI… and this vision must be adapted at regional and organizational levels." (P11)
			Better integration of AI	"If we integrate more, the results will be better." (P13)
	Health system structure and stability	Establishing risk management and oversight systems for AI in healthcare	Risk management system for AI in healthcare	"The whole regulatory approval system is based on risk reduction. You need a system for risk management." (P13)
			Risk management by regulatory bodies	"The main criterion of regulatory bodies is risk management… you need a system for risk management." (P13)

			Management of residual risk in AI systems	“You need a system for risk management. It is not just risk reduction, but also management of residual risk.” (P13)
			Board oversight of AI implementation	“Give CIUSSS boards the mandate to oversee the effective implementation of AI in their institutions.” (P6)
		Strengthening leadership, coordination, and national collaboration for AI deployment in health	Close collaboration with companies	“Researchers who develop digital solutions are not necessarily trained in this field and need close collaboration with companies to develop these solutions. These companies are also committed to providing necessary support during implementation.” (P2)
			Training on principles of partnership	“Provide basic training on the principles of partnership.” (P2)
			Collaboration between industry, health institutions, and universities	“Industry has the funding and skills to develop products; health institutions have access to clients and understand clinical and administrative challenges; universities train qualified health professionals and have the independence needed to objectively assess risks and benefits.” (P8)
			Strengthening industry–university collaboration to scale innovations	“We need more exchange between industry and the academic sector to implement innovations on a larger scale.” (P11)
			Government participation in testing AI in real-life conditions	“If I can test my device in real-life conditions and improve it… that would be an ideal collaboration with the government.” (P13)
			Government–startup collaboration for AI pilots	“The government can collaborate with startups and give them a trial period with reimbursement… that would be an ideal collaboration.” (P13)
			Need for leadership and coordination in AI adoption	“There must be a leader or coordinator in the health system to bring different stakeholders together for adopting new AI technologies.” (P15)
		Improving workflows and reducing administrative burden	Enhancing hospital administrative efficiency with AI	“Most hospitals could save millions of dollars by improving their administrative systems with AI.” (P13)
	Legal and ethical framework	Protecting privacy and data security in AI-enabled health systems	Clear and comprehensive regulations to ensure data protection	“When it comes to regulations… AI experts have already thought about it and found ways to securely share data.” (P9)
			Developing regulatory frameworks to ensure data protection and build trust	“No solution will ever be completely risk-free… but it must evolve over time.” (P9)
			Regulations for secure and ethical access to clinical data	“We need regulations that guarantee secure and ethical access to clinical data for AI projects.” (P10)
			Regulations for data privacy in AI and health	“We need transparent regulations for data privacy and ethical use of AI in healthcare.” (P11)
			Ensuring patient privacy in AI use	“Securing data and protecting patient privacy must be a key consideration in implementing AI tools.” (P15)
			Preserving patient privacy in AI implementation	“Patient privacy must be protected in the implementation of AI tools in the health system.” (P15)

		Developing transparent regulations and oversight systems for AI control and evaluation	Clear and understandable regulations for all stakeholders	"Regulations must be transparent… scientists, the public, physicians, and all involved should be present in designing these regulations." (P9)
			Clear and comprehensive regulations	"Regulations must be understandable… those involved should participate in developing them." (P9)
			Enforcing laws to prevent misuse of AI	"We need regulations… to ensure that those who do wrong are punished." (P10)
			Clear ethical guidelines for AI use in health	"We need clear guidelines and directions for the ethical use of AI in healthcare." (P11)
			Health Canada regulatory guidelines for AI	"Health Canada is issuing guidelines on how to integrate AI learning tools into the regulatory system." (P13)
			Updating medical device regulations for AI	"We must change the laws on medical devices and AI… to keep up with the pace of technological progress." (P10)
		Defining legal and professional responsibilities in relation to AI tools	Ethical and responsible use of AI	"I think AI has a place in every field, if it is used ethically and responsibly." (P2)
			Guidelines for integrating AI into primary care	"It would be interesting to develop guidelines for integrating AI into primary care." (P12)
			Physician responsibility in using AI data	"The physician must always remain responsible for using AI data. Conceptually, there is no difference between physicians using AI outputs now and in the past." (P13)
			AI medical devices should not replace physicians	"AI medical devices do not replace physicians… the physician must always remain responsible for using the data." (P13)
			Human interaction alongside AI tools	"We need human interaction… but supported by tools in the background that can analyze data quickly." (P10)
		Integrating ethical and human-centered principles in AI development and application	Regulatory and ethical frameworks	"Regulatory and ethical frameworks for AI in health." (P4)
			Ethical considerations (confidentiality, risks, etc.)	"Ethics (confidentiality, risks, etc.)." (P6)
			Ensuring compliance with ethical standards	"Respect for ethical standards." (P7)
			Developing ethical regulations for AI in health	"We need clear ethical regulations and guidelines for AI in healthcare." (P11)
			Human-centered approach in AI development	"Those who think about humans and their emotions should be involved from the beginning, not years after the technology has been developed." (P14)
			Patients' right to choose AI use in healthcare interactions	"Patients have the right to decide whether they want AI to be used in their appointments or interactions with health professionals." (P15)
			Establishing ethical and legal frameworks for AI regulation	"Policymakers must create ethical and legal frameworks to regulate the use of AI in health." (P15)
	Public acceptance and stakeholder engagement	Cross-sectoral and interdisciplinary collaboration among stakeholders	Collaboration to identify and prevent AI bias	"To identify and prevent the risks of bias in AI models, early collaboration between engineers, physicians, and ethicists is essential." (P1)
			Working groups with diverse stakeholders	"Consultation table or working group with diversity of perspectives/stakeholders." (P1)
			Ideal collaboration model	"Ideally, these three reinforce each other: universities provide independence and perspective to protect the public; industry provides funding and production logic; and

				health institutions are essential actors for testing, implementing solutions, and collecting relevant data." (P6)
			Collaboration of all parties	"Collaboration of all parties." (P6)
			Collaboration among all stakeholders	"Collaboration among all stakeholders." (P8)
			Collaboration among physicians, companies, and AI specialists	"We need to collaborate with physicians and companies to develop useful tools." (P10)
			Interdisciplinary collaboration in AI tool development	"We need collaboration between physicians, companies, and AI specialists… so that tools meet the needs of all stakeholders." (P10)
			Interdisciplinary collaboration for AI integration	"Interdisciplinary collaboration and training between health professionals and engineers is essential." (P11)
			Organizational commitment to interdisciplinary collaboration	"Every organization or every level of organization must allocate the necessary time and space to collaborate with others." (P14)
			Need for interdisciplinary and cross-sectoral collaboration	"Interdisciplinary and cross-sectoral collaboration will only happen if time is dedicated to it." (P14)
			Collaboration between AI developers and medical professionals	"Those who develop new technologies must meet with medical professionals to discuss how to create added value in the system." (P15)
			Collaboration between AI developers, medical professionals, and patients	"Developers of new AI technologies must meet with medical professionals and patients to ensure these tools create added value and are well accepted in society." (P15)
			Collaboration between developers, policymakers, and health professionals	"Developers, policymakers, and health professionals must collaborate from the outset to ensure AI tools meet the needs of the health system." (P15)
		Inclusive stakeholder engagement in health policy and technology development	Political will and stakeholder participation	"Political will and participation of all stakeholders (including citizens/patients)." (P1)
			Stakeholder participation from the outset	"Participation of all stakeholders from the very beginning." (P3)
			Involvement of engineers and patient-oriented experts in AI development	"You need an engineer, but also people who think about future concerns and patient well-being from the outset." (P14)
			Involvement of health professionals in AI development	"Health professionals should also be involved in technology development… this will make adoption faster among professionals." (P14)
			Participation of patients, health professionals, and policymakers in the development and implementation	"The development and implementation of new AI tools must involve patients, health professionals, and policymakers." (P15)
			Involvement of small groups of citizens	"Involve a small group of citizens and patient partners to prevent people from dropping out during development." (P2)
			Need for public participation in data governance and regulation	"We must be more aware of how our data is used and be involved in data governance… to combine expert perspectives with those of ordinary people." (P9)
			Involving ordinary citizens in AI decision-making and strategy design	"Denmark involved ordinary citizens in its AI strategy… and that led to the success of their five-year plan." (P9)

		Public education and awareness for AI acceptance	Clarifying AI benefits and risks to raise public awareness	“Let’s be transparent about risks and also about benefits, because both individuals and society will benefit.” (P9)
			Raising awareness about individuals’ rights regarding data use	“Some people want all the information, others only part of it… let people choose for themselves.” (P9)
			Developing educational frameworks for digital literacy and AI understanding	“Let’s try to educate people… so that instead of trusting ‘Dr. Google,’ they find accurate information and reliable sources.” (P9)
			Clarifying AI applications to avoid misconceptions	“Explain to people what you want to do and why… so they do not think the goal is to monitor them.” (P10)
			Public education on AI to prevent misunderstandings	“Explain to people what you want to do and why… so the purpose of AI is not misunderstood.” (P10)
			Public information campaigns on AI risks and benefits	“The Ministry of Health should raise public awareness of AI risks and benefits through campaigns.” (P11)
			Public campaigns on AI tools’ risks and benefits	“Public education campaigns must inform people about the benefits and risks of AI tools.” (P11)
			Patient awareness of AI tools	“If patients do not know what these tools do, professionals will not move toward using them.” (P14)
			Awareness of AI capabilities in health	“People need to be aware of the accessibility and capabilities of AI in health to be able to use it.” (P15)
			Appropriate AI training for stakeholders	“Proper AI training for stakeholders (professionals, users, etc.).” (P4)
		Enhancing transparency, public trust, and social participation in digital health governance	Prioritizing stakeholders’ considerations and consent	“Priority must be given to the specific considerations (and consent) of all stakeholders.” (P1)
			Community commitment	“Community commitment.” (P8)
			Trust in AI systems for emotion analysis	“People must know that machines can read their emotions… they must trust the system to interact with it.” (P14)
			Need for consent and clear understanding in AI emotion analysis	“Machines can read people’s emotions and physiological changes, but there must be clear consent and understanding from users.” (P14)
	Monitoring and effective evaluation	Developing evaluation, monitoring, and safety tools for AI use	Tools to measure AI impact	“Tools to measure the impact of AI on the quality of care and services.” (P4)
			Continuation of technology assessment institutions	“Institutions responsible for technology assessment (in Québec, INESSS, UETMISSS, etc.) must continue their work.” (P7)
			Continuous monitoring and evaluation	“Independent monitoring of outcomes.” (P8)
			Continuous monitoring of AI processes to ensure proper and ethical use	“We need specialists to monitor the use of AI… analyze what AI does and ensure it is beneficial for humans.” (P9)
			Ensuring AI safety for patients	“I want this system to be safe and useful for me as a patient.” (P10)
			Caution in using AI in mental health, especially suicide risk prediction	“We must be cautious in using AI in mental health, particularly in tools that predict suicide risk.” (P11)

			Validation of AI through medical examinations	"Honestly, if an AI can pass medical exams every year like a human resident, we are testing the same level of cognition." (P13)
		Monitoring and validation of AI applications in healthcare	Transparency in AI development process	"If the process is open, if everyone knows how it was developed and understands the pros and cons of each technique… there will be greater adherence across different technological development pathways." (P14)
			Practical testing of AI tools	"After developing a new AI tool, it must be tested in practice to determine usability and cost-effectiveness." (P15)
			Real-world testing of AI tools before implementation	"New AI tools must be tested in real-world scenarios to assess their safety, efficiency, and cost-effectiveness before large-scale deployment." (P15)
Financing & Incentives	Resource allocation and budgeting	Developing effective financing mechanisms for AI innovation	Rapid financing for AI projects	"We need seed funding for rapid financing to demonstrate the feasibility of AI projects." (P11)
			Dedicated funding for AI innovation centers	"Innovation centers do not have sufficient resources; we need dedicated funding and support." (P11)
			Support for publicly funded AI innovations	"In Norway and Ontario, they had systems where publicly funded innovations had free entry for three years." (P13)
	Economic evaluation	Cost–benefit evaluation of AI	Cost–benefit assessment prior to AI implementation	"New AI tools must be evaluated for cost–benefit before implementation." (P15)
Resource Generation	Infrastructure and Technical Equipment	Integrating infrastructure for effective AI adoption	Interoperability between systems for integrated data management	"Systems must be able to communicate with each other so that data management becomes more integrated for physicians." (P12)
			Integration of digital tools (e.g., health trackers) with electronic health records	"We must connect digital tools like health trackers directly to electronic health records to create better integration." (P12)
			Linking electronic health records with AI tools	"Electronic health records and AI tools must be connected." (P12)
			AI tool integration without retraining	"Once it is completed, these tools can be integrated like other tools that do not require retraining." (P13)
			Need for a coordinator for AI integration in the health system	"Someone must coordinate the integration of new technologies into the health system, such as the integrated health and social service centers in Québec." (P15)
		Enabling virtual care with AI tools	AI-assisted tools for virtual physical examinations	"We need advanced tools, such as AI-enabled cameras, to support virtual physical examinations." (P12)
			Improving physical examinations in virtual care with AI support	"If physical examination with greater AI support is better integrated into virtual care." (P12)
			Digital assistants for clients and physicians	"Provide digital assistants capable of guiding clients, and others that can help physicians stay updated on new knowledge and policies." (P8)
			AI chatbots for psychiatric support	"Launching a chatbot for psychiatry… it will be hard to test properly, but it could be helpful." (P13)
		Developing sustainable and	Development of open-access systems	"Ensure the development of open-access systems." (P5)
			Proven and efficient technologies	"Proven and efficient technologies." (P7)

		adaptable infrastructure	Maintenance and support by reliable providers	“Maintenance and support by reliable providers.” (P7)
			Adapting systems to technological progress	“We must adapt the system to the pace of technological progress… so that tools remain effective and practical.” (P10)
	Information resources	Ensuring confidentiality, transparency, and user consent in health data use	Informed consent and data confidentiality	“Participants must be informed about AI use, give their consent, and all measures must be taken to preserve the confidentiality of personal data.” (P2)
			Ethical use of data	“Ethical use of data.” (P7)
			Public participation in data governance	“We must participate in data governance… so that ordinary people’s perspectives are combined with experts’ views to provide solutions for data management.” (P9)
			Personalized patient access to data	“Some people want immediate access to their data as soon as it is available… others prefer to share their data only with their physician.” (P9)
			Responsible data access	“Facilitate responsible, ethical, and sustainable access to data (clinical encounter records and patient data).” (P5)
		Secure and transparent data management in digital health systems	Organizing data in health institutions	“Organizing data and information within health institutions.” (P4)
			Developing solutions for data management to ensure user security and transparency	“We must be more aware of how our data is used and participate in data management… to combine ordinary people’s views with those of experts.” (P9)
			Creating safer systems for data sharing without compromising privacy	“Secure data sharing is key… we must develop stronger methods to ensure security while maintaining collaboration.” (P9)
		Strengthening clinical research through diverse and high-quality data	Facilitating researcher access to data	“Health institutions can facilitate researcher access to information. I am cautious about industry involvement; I think we can perform well without it.” (P5)
			Greater trust in academic research compared to private industry	“I trust academic research more… when research is conducted in universities, it carries less risk compared to private industry.” (P9)
			Use of diverse data to better understand diseases	“We must look at more data and more diverse data because diseases are not one-dimensional.” (P10)
			Collecting more data from remote regions via virtual research	“We plan to collect more data from remote regions through virtual research projects.” (P12)
			Developing tools to securely and accurately analyze patient data for clinical research	“We must find ways to securely share data… and use AI to analyze it in a useful and ethical way.” (P9)
		Validating and ensuring accuracy of input data in AI systems	Ensuring data transparency	“Ensure transparency of data and results.” (P5)
			Ensuring accuracy of data input into AI systems	“Both patients and health professionals must ensure the accuracy of data provided to AI systems.” (P15)
			Ensuring accuracy of data input into AI systems	“Both patients and health professionals must ensure the accuracy of data provided to AI systems.” (P15)
	Human resources	Training and capacity-building of primary care providers in AI health applications	Introductory training prior to implementation	“Provide introductory training before deploying technological tools.” (P2)
			Developing educational approaches for physicians and the public to increase AI acceptance	“We must raise awareness and educate people… a differentiated level of understanding must be created.” (P9)

			Training physicians on AI for better decision-making	“We need to train physicians on AI so that they can ask the right questions and get the right answers.” (P10)
			AI training for physicians	“We must train physicians on the use of AI… so that they can trust the system and make better decisions.” (P9)
			Helping physicians ask the right questions with accurate data	“We must help them ask the right questions with the right data to reach the right answers.” (P10)
			AI education in medical curricula	“We still do not have such training in regular medical curricula… this training is essential for being able to ask the right questions.” (P10)
			Continuing professional education on AI for providers	“We need continuing professional education on AI for healthcare providers.” (P11)
			AI education in medical student programs	“We must educate students by integrating AI into their training programs.” (P11)
			AI training for health professionals	“It is important to train people in the system who use AI or technology so they understand how it works.” (P15)
			Training users and professionals for AI interaction	“Health professionals and users need training and awareness on how to interact with AI systems.” (P15)
			Training and empowering health professionals and users in AI interaction	“Health professionals and users need training and empowerment to properly interact with AI systems.” (P15)
			Educational materials for virtual care guidance	“Maple provided me with educational documents that specified what I could and could not do in virtual care.” (P12)
		Developing interdisciplinary skills at the health–technology interface	Need for bilingual experts in AI and medicine	“We need bilingual experts who understand both AI and the clinical domain to bridge this gap.” (P10)
			Interdisciplinary training programs for AI integration in health	“Interdisciplinary training programs where health professionals and engineers work together are the solution.” (P11)
		Reducing provider resistance to AI-related changes	Reducing resistance to change	“No resistance to change in clinical environments.” (P8)

Challenges of AI implementation in Iran's PHC

Table S3. Challenges of AI implementation in Iran's PHC

PCET	Main Theme	Sub Theme	Code	Quets
Stewardship	Health policy and governance	Awareness and attitudes of policymakers toward new technologies	Cultural challenges and lack of belief in evidence-based management	"There is a weak culture and insufficient belief in in-depth analyses and evidence-based management." (P2, P17)
			Lack of understanding of technological transformation and the importance of AI in the health system	"Our first challenge is that the health system unfortunately does not realize that this paradigm shift has occurred, and that AI technology is truly a game changer." (P20)
			Lack of understanding and alignment with the Fourth and Fifth Industrial Revolutions	"We do not realize… we think it's just an article or a hot paper, while in fact we are already going through a major part of the Fourth Industrial Revolution, and perhaps within a decade they will say the Fifth Industrial Revolution." (P20)
			Lack of technological vision in policymaking and data analysis	"The Ministry of Health does not allow proper access, processing, or analysis of data due to lack of national consensus and lack of understanding of the transformation. The mindset of policymakers is still rooted in the First and Second Industrial Revolutions." (P20)
			Lack of national consensus and traditional outlook of policymakers	"There is a lack of national consensus and inadequate understanding of the transformation, with policymakers' mindsets still rooted in outdated structures from the First and Second Industrial Revolutions." (P20)
		National strategy and strategic planning	Lack of a clear maturity model for AI implementation	"The absence of a defined AI maturity model is a serious challenge. For example, which part of our primary care system should we prioritize for smart transformation?" (P20)
			Lack of a comprehensive national AI strategy	"The lack of a realistic national strategy shows we do not truly understand what is happening. A national strategy should define the AI maturity model. Simply equipping universities with computers does not mean we are moving toward AI." (P20)
			Impulsive decision-making and lack of systematic planning for AI applications	"I find this issue dangerous… many actions in our country have not reached desirable outcomes because they were impulsive and poorly planned." (P25)
			Lack of transparency in goal-setting and absence of a clear smartization strategy	"At present, we don't know how to start the process… the main issue is that we do not know what exactly we want, and this is a serious problem." (P26)
			Losing opportunities due to delays in AI implementation	"We are missing the honeymoon period during which we could have fully benefited from AI. If we start too late, we may only face its long-term side effects without reaping the early benefits." (P28)

		Smart governance challenges	Lack of effective governance for managing AI applications	"The main issue with the AI system and its use—beyond the classic realm of public policy—is governance and the kind of transformation it can bring to people's lives." (P25)
			Large gap in smart governance compared to advanced countries	"In fact, the 2020 indicators, the official report of the Administrative and Employment Affairs Organization, comparing countries across six governance dimensions, show how far behind we are from OECD countries. We were supposed to reach the 2025 vision, at least in health, economy, and science and technology, but apart from slight progress in government effectiveness, we are far behind in other indicators." (P28)
			Flawed approaches including haste, missed opportunities, and fascination with technology	"Since the Qajar era, we have either rushed and wasted opportunities, or fallen into uncritical fascination with Western technology—almost like a curse—swinging between complete rejection and total infatuation." (P31)
			Weakness in smart governance indicators compared to advanced countries	"In terms of governance, our overall public governance situation is not good. The 2020 indicators, based on the official report of the Administrative and Employment Affairs Organization, comparing countries across six governance dimensions, show we are significantly behind." (P28)
	Health system structure and stability	Managerial instability and frequent changes	Incomplete implementation of projects due to short-term political goals	"Sometimes projects are launched just to be inaugurated under the name of a specific minister, even if they are not fully prepared, resulting in incomplete implementation." (P21)
			Lack of continuity due to managerial turnover	"The next official who takes over does not support the previous projects, since any achievements would be credited to their predecessor." (P21)
			Failure in large-scale projects	"Experience shows that in large-scale programs we have not been successful. Just look at our five-year plans… we need to start with smaller projects and build capacity before applying AI at scale." (P30)
			Preoccupation with daily crises	"In our country, we have a developmental paradigm, but in practice, managers are absorbed in daily crises. They say: 'I don't care what the experts discuss; on Monday morning I must solve my immediate problems.'" (P25)
		Weak managerial structures and infrastructures	Structural differences in health service delivery between Iran and other countries	"In Iran, services are divided into health and treatment, while in many countries primary care covers all services up to specialized medicine. This structural difference affects how AI can be applied." (P19)
			Complexity of defining AI use-cases	"First define the use-cases; then outline the features that distinguish them from other areas of health. Without clear packages, AI requirements cannot be identified." (P19)
			Lack of merit-based selection of managers	"Leaders should be selected based on merit and competence, not political alignments." (P21)
			Weak health indicators and discontinuity in program effectiveness	"Unfortunately, our health indicators are not strong and improvements are often temporary, as each new manager focuses on a different short-term priority." (P21)

			Mismanagement of practical issues	“Unless we establish proper governance, AI implementation in practice will remain unattainable.” (P32)
			Mismanagement of practical issues	“We are still stuck at the basics of management—AI requires much more advanced capacities.” (P32)
		Weak national and international coordination	Lack of national coordination and concerns over data confidentiality	“There is no national coordination. Issues like data confidentiality are not addressed—if we don’t process our own data, foreign actors will do it for us.” (P20)
			Lack of integration into international AI systems	“AI is inherently international; its equipment, algorithms, and datasets cannot be restricted domestically. Without integration, we cannot learn how best practices in PHC are applied elsewhere.” (P20)
			Lack of coordination between private and public sectors	“In Tabriz, a private company develops new technology while in Kerman the public sector works separately. There is no alignment between private and public initiatives.” (P24)
			Lack of coordination among institutions	“At the policy level, the absence of comprehensive laws and inter-agency coordination has hindered the effective use of AI.” (P25)
			Lack of strong coordination structures	“We have some intersectoral structures, but they are not sufficiently strengthened.” (P28)
		Lack of infrastructural and educational readiness	Dependence on foreign technologies and risks of sanctions	“If AI becomes part of our health and education systems, sanctions or disconnection from global technologies could paralyze all our efforts.” (P26)
			Absence of effective presence of the health system in digital space	“Our health system has little presence in digital platforms, preventing us from intelligent monitoring and engagement.” (P26)
			Insufficient readiness of universities for AI training	“Universities are not yet prepared to provide the necessary training for AI-related skills.” (P30)
			Lack of organizational motivation for AI projects	“Organizations often lack motivation to pursue AI projects; without strong incentives, initiatives are abandoned.” (P22)
			Lack of support for startups and new ideas	“Innovative ideas face difficulties in advancing due to insufficient support; startups often withdraw.” (P22)
			Outdated health network and skills	“Neither our health network nor our skills have been updated to match the needs of AI adoption.” (P31)
	Effective monitoring and evaluation	Weaknesses in oversight and performance evaluation	Lack of effective monitoring in AI project implementation	“If policies exist only on paper without supervision and inspection, they may not succeed—similar to previous Ministry of Health directives, since hospitals were more concerned about staffing needs.” (P23)
			Absence of regulatory mechanisms for AI monitoring	“The lack of regulatory mechanisms to evaluate and monitor enacted laws is a serious barrier to AI development in the health system.” (P25)

		Weakness in program effectiveness monitoring	Serious deficiencies in disease surveillance and inefficiency of current methods	"Currently, our disease surveillance system has severe deficiencies, and no matter how much we invest, significant improvement cannot be achieved. But with a fully intelligent system, many of these shortcomings could be resolved." (P26)
		Error tracking and decision-making accuracy assessment	Incorrect diagnoses	"There is a risk of incorrect diagnoses." (P1, P16)
			Risk of inaccurate decision-making	"There are risks of incorrect or misleading decision-making." (P1, P16)
			Complexity of decision-making for healthcare providers	"In our health system, family physicians or health workers must make decisions based on numerous interrelated variables. This often leads to excessive complexity, which current systems cannot manage effectively on their own." (P25)
	Legal and regulatory framework	Responsibility and accountability of AI	Challenges of automated decision-making without explainability	"In some cases, instead of humans making decisions, we may rely on automated decision-making systems. That is where the explainability of AI becomes a critical issue." (P18)
			Lack of legal and ethical accountability for AI	"Ultimately, as a physician, I am accountable for my diagnosis. The question is whether AI can bear such responsibility." (P19)
		Legal restrictions and barriers to AI development and application	Restrictive laws for AI development	"Given the current laws—some of which date back many years—progress in AI and technology in general is nearly impossible unless regulations are bypassed." (P22)
			Burdensome regulations for the private sector	"To obtain a license, for instance, companies must meet heavy security and insurance requirements, which only two or three firms out of dozens can realistically fulfill." (P22)
			Legal obstacles to AI progress	"With the current regulations, it is impossible to make progress. When we attempted to run a project, we simply couldn't move forward under existing laws." (P22)
			Legal limitations in AI project implementation	"With the current regulations, it is impossible to carry out AI projects in this country." (P22)
			Legal and regulatory barriers to AI use	"There are numerous legal and regulatory barriers to the use of AI in the health system. These need to be identified and addressed for AI to be effectively utilized." (P25)
		Incompatibility of laws and lack of legal standards	Lack of legal and ethical guarantees	"Health services are highly sensitive. You cannot prosecute or punish an AI tool for making a mistake—AI inherently does not provide guarantees." (P19)
			Outdated laws not aligned with AI technologies	"Current regulations are outdated and incompatible with the pace of AI advancements." (P22)
			Absence of comprehensive laws	"At the policy-making level, the lack of comprehensive, clear legislation and poor coordination among institutions prevent the effective use of AI." (P25)
			Lack of adequate legal and ethical frameworks	"The legal and ethical foundations necessary for AI use in healthcare are not yet in place." (P27)
			Lack of comprehensive legal documents for AI	"When starting projects, we realize that legal and ethical frameworks are still missing, which hinders implementation." (P27)
			Absence of legal protocols and standards	"In current clinical care protocols for chronic diseases such as diabetes and hypertension, online or remote services are not yet permitted." (P32)

	Public acceptance and stakeholder engagement	Institutional and social resistance to technology adoption	Social resistance against new technologies and data transparency	“There are strong social resistances to transparency-enhancing technologies, as they can prevent corruption, and many groups and stakeholders oppose them.” (P22)
			Institutional and administrative resistance to new technologies	“The Ministry of Health resisted electronic prescriptions for over a year, even issuing official letters instructing universities not to cooperate with insurers.” (P23)
			Resistance to transparency due to vested interests	“We faced strong opposition from the Food and Drug Organization and pharmacists because transparency technologies threaten corruption and block certain practices.” (P23)
			Resistance at specialized and managerial levels	“Resistance mainly occurs at secondary and tertiary levels of the health system.” (P23)
			Lack of collective mobilization for smart health reforms	“The real challenge is creating a wave that everyone joins rather than stands against. This requires careful planning.” (P26)
			Structural resistance to digital transformation	“In our country, many universities, national, and ministerial structures strongly resist digital transformation, which hinders essential health reforms.” (P26)
			Resistance to necessary changes for AI implementation	“There is a reluctance to move toward AI adoption.” (P28)
			Cultural resistance against new technologies	“There is a culture of resistance to technology across various levels of the health system, including governance structures.” (P20)
			Resistance to altering existing systems	“We already have programs for expanding the current system (like insurance), but we avoid changing them altogether.” (P23)
		Lack of public awareness and digital literacy	Low digital literacy and awareness about AI	“One of the main problems is people’s low digital literacy, which has not been sufficiently addressed.” (P25)
			Lack of awareness among service recipients	“At the primary care level, both providers and recipients of services are not adequately informed about AI.” (P21)
			Lack of public awareness of AI benefits and health records	“People are reluctant to provide their information because they are unaware of the benefits of AI and electronic health records.” (P21)
		Public trust and social concerns	Spread of misinformation affecting public health	“Unfortunately, we are exposed to fake information, which negatively impacts public health and creates complex challenges.” (P25)
			Lack of mechanisms to counter misinformation	“The absence of proper mechanisms to identify and combat misinformation is a serious challenge.” (P25)
			Trust risks	“Trust risk.” (P1)(P16)
			Policymakers’ lack of trust in AI	“Policymakers are skeptical about implementing AI at scale, as localized experiments might undermine broader credibility.” (P18)
			Declining public trust due to surveillance concerns	“People believe their SMS or online activities are being monitored, which reduces their willingness to share information.” (P28)
			Lack of social and political participation in AI governance	“Many national initiatives have failed due to lack of social and political participation—an essential element of good governance.” (P25)

		Social participation and negative social implications	Decline of health system users due to competition from virtual platforms	"Currently, only about 20% of people use the health network. With new virtual platforms offering better services, we risk losing most of our users." (P28)
			Fraud and misuse of AI and health data	"We must remain cautious, as fraudsters and abusers of health data may exploit AI alongside legitimate users." (P29)
			Potential misuse of AI for surveillance and social control	"Political systems could use AI for social control—for example, monitoring citizens via facial recognition or restricting freedoms under the pretext of security." (P20)
Financing & Incentives	Resource allocation & budgeting	Inefficient allocation of resources	Limited financial resources for AI development	"Funding depends heavily on scale; it can range from 500 million tomans up to 5–6 billion tomans if we bring in national datasets." (P18)
			Inefficiency in allocating health financial resources	"We have a serious problem in optimally allocating health financial resources, and this needs to be addressed through stronger research networks." (P25)
			Lack of dedicated budgets for AI investment	"One of the serious challenges is that policymakers still see AI as a luxury rather than a necessity, which prevents allocation of adequate budgets." (P25)
			Insufficient resources for AI implementation	"We are facing financial challenges, and the necessary funds for AI projects must be provided more quickly." (P27)
			Financing barriers delaying implementation	"Financing challenges can delay implementation; bold proposals may succeed in some regions, but broader governmental uptake is uncertain." (P24)
			Rapid technological updates creating financing gaps	"With frequent updates, like multiple new versions of ChatGPT in just a year, financing becomes increasingly challenging." (P24)
			Lack of funding and lengthy decision-making processes	"Decision-makers often delay by saying we lack money, resources, or time, pushing projects to future plans." (P27)
		High investment costs	High initial investment costs	"High initial investment costs." (P1)(P16)
			High costs for AI implementation	"We face significant financial challenges, and the high costs required for implementation must be addressed quickly." (P27)
		Low willingness to invest	Limited willingness to invest	"Some policymakers consider AI a luxury and are therefore less willing to invest in it." (P25)
			Lack of private sector motivation due to low ROI	"The private sector needs a clear return on investment—short, medium, or long-term—before committing funds." (P21)
			Reluctance of insurers to fund preventive services	"Insurers are reluctant to invest in preventive services, fearing that longer life expectancy will increase long-term costs." (P28)
	Payment mechanisms	Inefficiency of payment systems	Inefficiency of uniform insurance schemes	"We face a serious challenge because the insurance system applies the same premiums to very different groups, which cannot meet their diverse needs." (P25)
			Lack of payment mechanisms for AI-based software	"Out of 46 e-prescription software systems, their share is below 8%... and social resistances further hinder adoption." (P22)
		Complexity in service valuation	Lack of new tariffs for AI technologies	"The tariff system has not introduced AI-specific codes, delaying the integration of new technologies into the health system." (P24)

			Complex valuation by Supreme Insurance Council	"Insurers question costs during negotiations, leading to delays and inconsistencies in approving tariffs for new AI services." (P24)
Resource Generation	Infrastructure and Technical Equipment	Immaturity of infrastructure	Lack of maturity in AI infrastructure	"We have not yet reached the required maturity in infrastructure for AI use." (P24)
			Lack of infrastructure for data analysis	"Our information system has many problems, including lack of infrastructure, poor system coordination, and inadequate analytical capabilities, which hinder timely and accurate decision-making." (P25)
			Inadequate IT and hardware infrastructure	"Even at top universities in Tehran, IT infrastructure is insufficient; for example, family medicine projects face serious IT challenges." (P27)
			Lack of IT infrastructure for AI	"Even at top universities in Tehran, IT infrastructure is so weak that we face problems even in family medicine projects." (P27)
			Lack of electronic appointment systems	"In our clinics, patients crowd because we lack electronic booking systems like banks, mainly due to financial constraints." (P31)
			Reliance on low-quality systems	"The Ministry of Health continues to insist on using low-quality systems such as the SIB platform." (P32)
			Difficulties upgrading electronic to intelligent systems	"When we try to upgrade electronic systems to smart ones, the level of maturity is insufficient and problems arise." (P26)
			Inadequate data analysis capabilities	"Our system lacks proper tools for data analysis, and AI is not effectively used to support decision-making." (P25)
		Lack of coordination & integration	Lack of interoperability among local apps	"Different provinces and counties develop their own apps, but these AI systems cannot interact or exchange data." (P21)
			Lack of coordination across systems	"Our information system suffers from limited access, lack of coordination, and poor use of AI in decision-making." (P25)
			Lack of comprehensive & coordinated systems	"Absence of coordinated systems prevents timely and accurate decision-making." (P25)
			Lack of infrastructure for cross-system integration	"Due to lack of infrastructure, systems cannot connect, causing information gaps and inefficient decision-making." (P25)
			Lack of precise data fields for linking datasets	"Currently, fragmented databases lack precise fields to properly link information." (P26)
			Lack of integration among health IT systems	"Our systems lack integration and data quality is poor; this is a major challenge." (P27)
			Lack of clarity in integrating multiple systems	"It is still unclear whether our five main systems will ever be integrated." (P31)
			Lack of lab integration in data exchange	"We failed to integrate labs nationwide, e.g., daily reporting of basic test results is missing, while it is standard elsewhere." (P31)

		Technical limitations	AI more suitable for simple rather than advanced services	“Some argue that AI is more useful for simple services than advanced ones.” (P19)
			Heavy dependency of AI on hardware	“AI depends heavily on strong hardware; without it, efforts will be ineffective.” (P26)
			Shortage of advanced AI equipment in the market	“Even if funding is available, many AI hardware components are no longer accessible in the market.” (P26)
			Contradiction of AI with local technologies	“There is concern whether AI applications in PHC contradict local technological principles.” (P19)
	Information resources	Lack of integration & coordination of data	Fragmented data across institutions	“Some data are with medical universities, some with social security, and some with the armed forces; there is no unified aggregation.” (P22)
			Problems aggregating databases	“There are serious challenges in aligning and aggregating databases.” (P24)
			Separate storage of data hindering integrated analysis	“Different datasets are stored separately, and current systems cannot provide unified access, leading to gaps in decision-making.” (P25)
			Absence of comprehensive analytic systems	“Data are stored separately without integration, making analysis and decision-making very problematic.” (P25)
			Lack of linking fields across databases	“Databases are fragmented and often lack precise fields for linking.” (P26)
			Lack of effective integration among health IT systems	“Our systems lack interoperability and data quality is poor; this is a major challenge.” (P27)
		Deficiencies in standardization & data management	Lack of data standardization	“There are challenges in ensuring consistent formats for data collection.” (P18)
			Excessive forms discourage participation	“There are too many forms and questions; even though necessary, people rush through them as a routine.” (P21)
			Uncoordinated data registration influenced by financial factors	“Financial and structural issues lead to uncoordinated data registration across platforms.” (P27)
			Poor use of generated data due to a lack of management	“Large amounts of data are generated, but they are not managed effectively.” (P27)
			Non-standardized data registration processes and fragmentation across health IT systems	“…Data registration in other software platforms often shifts toward certain sections due to financial structures, which prevents a coherent and standardized process from taking place overnight.” (P27)
			Underutilization of large volumes of health data due to lack of proper management	“…We have a large amount of data being generated, but many other datasets cannot be processed effectively. Think about what happens if we fail to manage and use them properly.” (P27)
			Weaknesses of health information systems & databases	“Information systems and databases are fragile and do not meet required standards.” (P2)(P17)

			Challenges in managing large, complex datasets	"Handling decades of patient records is problematic due to both data volume and limited analytic capacity." (P19)
			EHR weaknesses as a lesson for AI	"The electronic health record has faced serious challenges; we should learn from these issues for AI." (P30)
		Poor quality, accuracy & reliability of health data	Low data quality	"Data quality is poor, and there is no simple way for trust validation." (P26)
			Lack of proper data validation systems	"There is no effective trust validation system for incoming data." (P26)
			Lack of proper data validation systems	"…Currently, there is no simple way for trust validation. With AI we might be able to address these shortcomings in a more intelligent way." (P26)
			Inaccuracy of AI outputs	"AI results are often inaccurate, which risks discrediting entire projects." (P18)
			Lack of filtering for input data	"We lack proper filters to validate data entered by doctors or users." (P22)
		Restricted access to data	Difficulty accessing big & comprehensive datasets	"Access to large datasets is crucial for AI, but currently difficult." (P18)
			Problems accessing health information systems	"Access to health information systems is a major challenge." (P25)
			Limited information access	"Our system faces limited access to information, poor coordination, and weak AI support for decision-making." (P25)
		Security & confidentiality concerns	Risk of confidential data leakage	"Risk of accessing and leaking confidential information." (P1)(P16)
			Cybersecurity threats & hacking risks	"Hackers and foreign adversaries pose serious risks, slowing down bold AI initiatives." (P23)
			Privacy & safety concerns in AI	"In healthcare, confidentiality and patient safety issues hinder smooth adoption compared to other industries." (P25)
			Security challenges in data access	"Sometimes there are security issues with data access." (P26)
			Misuse of data by foreign platforms	"Foreign platforms can use our scattered data to predict behavior and set their own policies." (P28)
			Data security as a key challenge	"Data security is one of the main challenges." (P32)
		Explainability & interpretability issues	Lack of explainability in AI models	"Machine learning models, especially neural networks, often work as black boxes, reducing trust among policymakers." (P18)
			Misalignment in interpretation of data	"Definitions and standards used in data collection may differ, leading to inconsistent interpretations." (P21)
			Lack of transparency in AI algorithms	"There are major concerns about transparency in AI algorithms used for decision-making." (P22)
	Human resources	Unequal distribution of the workforce	Workforce shortages and variation in skills/attitudes	"We face both shortages and differences in scientific knowledge, skills, and attitudes among staff." (P21)

			Looming retirement crisis	"In the next three years, almost 80% of our staff will retire; this is a major workforce crisis." (P22)
			Shortage and inequitable service provision	"Shortages in human resources already exist, and in the future equitable service delivery will be even harder." (P26)
			Shortage of nurses & doctors in deprived regions	"We constantly face shortages, especially in underserved areas where we lack nurses and doctors." (P29)
		Lack of specialized workforce	Weak scientific foundations for AI initiatives	"Our work must be based on solid scientific foundations, not just discussions." (P31)
			Lack of skilled staff to operate AI tools	"Few staff in our PHC or health system are truly capable of operating AI-based tools." (P26)
			Shortage of experts to launch/manage AI	"When it comes to PHC and AI in Iran, very few individuals are available to set up and manage such systems." (P27)
			Limited AI experience	"Experience with AI is minimal." (P2)(P17)
			Shortage of AI experts	"Very few AI experts remain in Iran; those available often emigrate quickly." (P18)
			Lack of AI-related specialties in academia	"Only a handful of people with AI-related expertise exist across our ICT faculties." (P27)
			Insufficient specialized staff to manage AI	"Even if an AI package exists, there are very few people in the country to run it." (P27)
			Limited training capacity for AI professionals	"We currently lack the capacity to train enough AI-related professionals." (P27)
		Lack of AI-related knowledge & skills	Limited basic knowledge of AI	"Basic knowledge of AI use in the health system is limited." (P2)(P17)
			Lack of trial-and-error culture	"We need to execute, test, and normalize technologies through trial and error." (P23)
			Lack of specialized AI skills	"Doctors lack skills such as coding in Python or R needed to use AI." (P20)
			Lack of mindset and readiness for AI	"Many experts cannot conceptualize AI-based processes to achieve results." (P21)
			Lack of awareness among service providers	"In PHC, both providers and recipients lack awareness about AI." (P21)
			Lack of training for users	"Neither the system nor regulations provided proper AI training for users." (P22)
			Insufficient awareness of proper AI use	"Many AI tools are underused because physicians and health workers lack training and awareness." (P25)
			Amateur/non-professional application of AI	"At the PHC level, AI is often used in an amateur or unprofessional way." (P30)
			Outdated health workforce skills	"We neither modernized our health networks nor updated our skills." (P31)
		Individual & professional resistance	Resistance due to lack of AI perspective	"People like me find it hard because our mindset is not AI-oriented." (P21)

			Scientific resistance to AI adoption	"Many experts cannot conceptualize AI-based processes to achieve results." (P21)
			User resistance to technological change	"Around 40% of people cannot adapt to new technologies, no matter what." (P22)
			Physicians' reluctance to use AI in decision-making	"When data are used, physicians may resist but AI can push them toward behavioral change." (P29)
			Traditional professionals rejecting AI-based processes	"Many experts cannot conceptualize AI-based processes to achieve results." (P21)
			Fear of job replacement by AI	"Resistance may occur when people fear being replaced by AI." (P30)
Service Delivery	Accessibility	Ineffectiveness of AI in remote areas	AI limitations in rural/remote settings	"AI alone can never solve my problems in rural and remote areas or in the first level of care." (P19)
		Inequitable access to services	Lack of insurance diversity leading to inequality in healthcare delivery	"We have a serious health issue: a uniform insurance system that applies equal premiums across groups. This has led to inequities, as those with greater needs receive the same as those with fewer needs. The lack of diversity in insurance coverage is a major challenge." (P25)

Requirements of AI implementation in Iran's PHC

Table S4. Requirements of AI implementation in Iran's PHC

PCET	Main Theme	Sub Theme	Code	Quets
Stewardship	Health policy and governance	Developing a national roadmap and strategy for AI implementation	A roadmap for proper and intelligent development	"A roadmap for proper and intelligent development." (P16)
			Developing a national AI model for health	"The first step seems to be having a clear and country-adapted model as a necessity." (P21)
			Necessity of a national AI strategy in the health system	"…If we can, with the legal basis I mentioned, establish a national strategy, we already have the general system, but we must take serious action on this…" (P25)
			Need for a comprehensive national AI strategy for health	"…We need a comprehensive national strategy for the use of AI in the health system. This strategy should cover all dimensions of AI, including medical, research, and managerial applications…" (P25)
			Necessity of a comprehensive and coordinated approach to AI	"…We must reach a comprehensive and coordinated approach for using AI in the health system. This should include appropriate policymaking, developing technical infrastructure, and enhancing knowledge and skills related to AI…" (P25)
			Developing a comprehensive and coordinated AI strategy	"…The use of AI must be based on a comprehensive and coordinated strategy that considers all aspects of this technology. It should be designed to effectively and efficiently improve the health system…" (P25)
			Developing a comprehensive national AI strategy	"…A national strategy must be developed for AI use, covering all aspects of this technology, to improve health services at various levels. This strategy should include clear goals, regulatory mechanisms, and continuous monitoring…" (P25)
			Developing macro-level documents and specific executive programs	"…Our executive managers in the health system—our senior leaders—must take a step forward and write the overarching document. Then gradually the sectoral documents and detailed programs will be prepared…" (P27)
			Establishing clear frameworks for AI implementation and transition management	"…We must create proper frameworks. Of course, this transition has challenges: people may resist change or fear being replaced. In any case, it should not be handled in a way that puts us in a vulnerable phase when another technology comes along…" (P30)
			Need for a clear roadmap and gradual preparation for AI in PHC	"…We must be careful that urgent recruitments and hasty actions without a proper roadmap and clear goals do not undermine the fragile existence of PHC. Appropriate groundwork must be prepared…" (P32)
			Designing efficient models to facilitate AI use	"…A model should be provided to individuals so they can achieve their own specific efficiency…" (P21)
			Necessity of effective AI governance to improve people's lives	"…The main issue with AI systems and their use is not only in the classical public policy sense; it is governance itself and the change it can bring to people's lives…" (P25)

			Creating planning systems and comprehensive AI strategies	"…We must seek to create systems that, through proper planning and comprehensive strategies, improve the use of AI. These systems must be designed to analyze data effectively and accurately…" (P25)
			Improving efficiency, management, and policymaking of AI in PHC	"…It is not just about using the advantages; it is also a necessity to improve efficiency and performance within the system, strengthen management, and enhance policymaking, particularly in PHC and health service networks…" (P27)
		Smart and Data-Driven Governance in Health	Transition to a data-driven system	"…If we understand this, our health system must be data-driven. Compared to three decades ago, we have gone through several waves of technology…" (P20)
			Need for smart governance to detect inequalities and support decision-making	"…Smart governance is essential for health in the 21st century. Our research shows that to assess inequalities and support decision-making—for example, by family physicians or health experts—AI can handle the complex interactions of numerous variables that humans cannot easily identify…" (P25)
			Creating a smart ecosystem and evidence-based policymaking	"…We need an ecosystem for health, where multiple actors can interact intelligently, and policymaking is evidence-based—leveraging evidence that AI can help generate…" (P26)
			Developing evidence-based policies and using research centers for AI development	"…Provided that we adopt proper ranking, principles, and evidence-based policymaking, alongside the use of research centers, we can achieve this…" (P30)
			The necessity of assessing the current situation before AI implementation	"…Do we have the capability, the skills, and the funding? We must assess the current situation; otherwise, having nothing prepared will bring no benefit…" (P31)
			Identifying opportunities, challenges, and timely intervention	"Identifying opportunities, challenges, and timely intervention." (P16)
			Managing AI adoption risks	"Managing the risks of AI adoption." (P16)
		Facilitating targeted and forward-looking policymaking for AI	Developing long-term and short-term planning for AI	"In my view, you should divide your plan into two parts: a long-term plan and a fast track. The short-term plan should showcase an interesting application derived from AI data analysis—something not easily obtained through traditional statistics—and demonstrate it. From a policy perspective, this requires building proper datasets." (P18)
			Revising health programs	"Therefore, it seems that in the field of health, scholars and experts should sit together to redesign the national health system program… not politically, but based on specific contextual needs that may exist temporarily." (P21)
			Need for convergence between planning and implementation in AI use	"…In our country, we have a developmental paradigm. As an executive, I say: I don't care what is said in theory—I must solve my problem by Monday morning. The real issue is that in implementation we face problems, and this gap between planning and execution must be addressed…" (P25)
			Developing a comprehensive program for software, human resources, and equipment	"…We need a new and comprehensive perspective on software, human resources, and the equipment required for AI, and stronger planning must be carried out." (P26)

			Establishing long-term plans for optimal AI utilization	"…If we are wise and truly make use of these tools, there may be initial costs in the short term, but in the long term—over decades—this will certainly result in major savings." (P27)
			Using a forward-looking approach to improve health systems	"…We must approach this with foresight. Mental and social health, physical indicators, food security—all these come together. You can then test whether this has helped or not…" (P28)
			Defining clear objectives for AI applications	"…What is this tool for? If you set up a session, what should our goals be? Do we have the capabilities, the skills, the funding?" (P31)
			Purposeful definition of AI applications	"…In my opinion, AI is definitely a tool. Almost all of us already use it in preparing papers or slides. But once it becomes a tool, we must identify clear goals for its use." (P31)
		Strengthening leadership, commitment, and national participation for AI implementation	Developing comprehensive long-term AI programs with expert involvement	"…We should set a general five-year framework, add annual summaries, engage experts, and ensure that within this framework, support is provided." (P21)
			Establishing think tanks and depoliticizing processes	"…Individuals, regardless of political affiliations, and those with academic expertise should be engaged in the process. Think tanks should be established to address the necessary challenges." (P21)
			Creating a National AI Council in the health system	"…Councils should be formed. If I were the Minister of Health, I would establish an AI Council in the Ministry." (P27)
			Establishing managerial and policy commitment to AI implementation	"…We need managerial commitment at the highest levels, as well as policy commitment. This must take root among senior leaders of the health system and the country." (P27)
			Establishing coordinating structures such as a Strategic AI Council	"The idea of an AI Council is very innovative." (P32)
			Selecting managers based on merit, not political affiliation	"…Elites should be chosen from the bottom up, based on merit, rather than being politically appointed." (P21)
		Institutionalizing innovative and flexible policymaking in the health system	Adapting policymaking to the characteristics of the new generation for innovative human development	"…Behaviorally, it is crucial to pay attention to Generation Z, with traits such as curiosity, creativity, and focus on value creation rather than mere entrepreneurship. These individuals will serve as both providers and recipients of health services. Without addressing their needs, PHC models will not succeed. A creative, dynamic, and hopeful human resource can generate value, wealth, and health." (P32)
			Necessity of improving policymakers' understanding of AI's transformative role in health governance	"…AI is not just advanced data analysis—it is the very playing field of governance in today's world. It is not merely a tool; it defines the arena itself. Policymakers must fully grasp this reality." (P32)
			Acceptance of modular thinking and system flexibility	"…Modularity and flexibility in PHC can be achieved through linking Iran's economy with the global economy." (P20)
			Separating politics from health services	"…Our service domain should be separated from politics. When politics is detached, political interventions will no longer directly impact health and healthcare delivery." (P21)
			Changing insurers' perspective towards healthy life expectancy and its economic effects	"…The important point is that people with healthier life expectancy can generate more wealth, and as a result, insurance contributions can increase because people live healthier lives." (P28)

			Changing managers' perspective on AI and telemedicine as facilitators	"It is essential to change managers' perspectives so that AI and telemedicine are recognized as facilitators of healthcare delivery at all levels." (P32)
		Leveraging global experiences for optimizing AI policymaking and implementation	Learning from other countries' AI experiences	"…We must fully utilize technology. Our people rely on us, and it is our duty to bring in the experiences of other countries as soon as possible, so that our citizens also benefit." (P24)
			Learning from successful digitalization initiatives	"…We must draw from successful digitalization experiences, such as in banking, and apply them to improve the health system." (P26)
			Using international experiences and successful models for AI implementation	"There is no need to reinvent the wheel! We should use the experiences of others." (P32)
	Legal and Regulatory Framework	Developing comprehensive legal and ethical frameworks for AI use	Developing legal and ethical frameworks for AI use	"…The safety debate in language and all these issues started in the 1990s and 2000s; we witnessed competitions one after another, and now it has reached models like GPT and ImageNet algorithms." (P19)
			Developing regulatory frameworks	"Regulatory frameworks for AI are influential, as they ensure the technology does not deviate from intended goals." (P22)
			Developing a comprehensive regulatory framework for AI	"Regulatory frameworks for AI are influential, as they prevent deviation from objectives and ensure individuals can operate within clear boundaries." (P22)
			Need for comprehensive and unified legislation for AI implementation	"We need a set of laws and regulations to properly advance these systems." (P23)
			Developing comprehensive legislation and oversight mechanisms for AI use	"…To use AI effectively, comprehensive and explicit laws must be developed to cover all aspects of this technology, along with mechanisms for monitoring and evaluating these laws." (P25)
			Ethical codifications	"Ethical codifications." (P16)
		Developing executive, legal, and juridical tools	Need to create legal and juridical tools	"One of my requests is that colleagues in the Ministry of Health network must provide the legal instruments for this." (P25)
			Developing legal and juridical tools for AI use	"…Appropriate legal and juridical instruments must be developed for AI use in the health system. Without these tools, utilizing AI in this sector will not be possible." (P25)
		Developing legal and ethical standards and protocols	Using standardized algorithms and methods in the health system	"…Having an algorithmic basis that can implement this model in a fully classical and classified way alongside the traditional system." (P21)
			Standardization and creating systemic interactions	"…We need standardization frameworks and regulations. I referred to both governance-level rules and some technical regulations, because technical standards are also necessary before we can fully engage with AI." (P22)
		Enhancing standardization and human oversight in AI implementation	Developing evaluation and monitoring standards for AI	"Systems to verify AI performance and precise standards for evaluating its effectiveness are essential." (P20)
			Necessity of human oversight in AI health processes	"…Naturally, AI services are still new, and health services are highly sensitive. There must always be a human supervisor engaged. You cannot simply blame or prosecute AI if it gives a wrong answer; from the start it has no guarantee in its outputs." (P19)

	Health System Structure and Stability	Phased and pilot implementation to facilitate AI adoption	Need to design pilot projects in regions with medium-level economy and infrastructure	"…At a medium level, moving from a global scale to a regional scale, this will happen. For example, the Ministry of Health began the golden thousand days of life initiative, similar to what many countries did, in places like Bandar Abbas, Zabol, and Zahedan near the Afghan border—practically, others followed us afterwards." (P23)
			Designing simple and feasible projects to reduce resistance and enhance readiness	"…A very simple initial design—for example, in Oman they monitor people's blood pressure in a way that reduces costs. We should do something simple as well. Also, when a country has already tested something, it makes it easier to move forward with higher-level steps." (P23)
			Implementing pilot projects and presenting results to policymakers to reduce resistance	"…We should do this and place the policymakers in front of a concrete example, showing that we have already implemented it in a region ourselves." (P23)
			Conducting pilot projects to assess challenges and capacities	"…We must carry out pilots—in a location, a university, a county, or a village. Your recommendations must be practical and applicable." (P23)
			Implementing large-scale pilot projects to evaluate and adapt new systems	"…It is necessary to study larger communities, not just a single family. For example, the entire city of Rafsanjan should go under this new method as a pilot." (P24)
			Need for pilot AI projects to test performance in specific areas	"…We are ready to test it in a specific place—in a province, with available health facilities—and check how the initiative progresses." (P29)
			Starting with small and gradual projects to expand AI use	"…We should start with small, problem-oriented projects and gradually move towards larger ones. You cannot just focus on one component like AI while neglecting other components of PHC that must work in harmony." (P30)
		Redesigning the health system structure for AI integration and efficiency	Integrating health analyses nationwide using AI	"…We should design a nationwide system so that we can be assured of having at least one unified analysis across the country." (P21)
			Reforming and upgrading Iran's health network through AI	"…Iran's health network, developed with great effort by respected scholars over the years, is not a bad system, but it needs reform." (P21)
			Expanding the health network system to comprehensive and universal services	"…We must shift our health network system towards comprehensive and universal health services. For example, in the Supreme Council of Health, they even added food security as part of its scope. In my analysis, this is the concept of 'One Health.'" (P28)
			Creating flexibility in systems	"…Modularity and flexibility in PHC can be achieved through linking Iran's economy with the global economy." (P20)
		Designing and aligning AI packages with real health system needs	Precisely defining AI application packages for implementation	"…You must define an AI package before looking for its requirements. The challenge is that AI is not a closed box—it soon enters wherever human intelligence is present." (P19)
			Defining standards and distinctive features for AI packages	"…AI will soon enter every domain where human intelligence exists, perhaps even more extensively. Before discussing requirements, first define the package. And to define a package, you must determine its distinctive features compared to other parts of the health sector." (P19)
			Designing AI systems suitable for simple services and underserved areas	"…Three key points: PHC services are supposed to be simple and basic; PHC is meant to serve deprived areas; and PHC must maintain health records for individuals and families. So we must

				see how AI can help in such simple services, especially in remote and underserved regions." (P19)
		Strengthening intersectoral collaboration for synergistic AI implementation	Inter-organizational collaboration	"Inter-organizational coordination is very important. We feel the need for certain regulations to clarify how this should be done, but they are lacking." (P22)
			Coordination between insurers and other organizations to create synergy in project implementation	"…From the insurers' perspective, although it may not look that way from outside, costs like paying for streptokinase after a stroke must be covered to prevent deaths. The financial system must stand behind such measures. If only policies exist without real commitment from hospitals and doctors, outcomes will fail, as seen in many prior Ministry of Health initiatives." (P23)
			Need for effective collaboration between healthcare providers and financing organizations to benefit from new technologies	"…If financing organizations demand AI, then certainly healthcare providers can move forward more effectively." (P24)
			Creating participation and convergence among different sectors to prevent resistance	"…We must act in a way that brings all groups together and prevents resistance. Such alignment requires genuine collaboration between different sectors." (P26)
			Coordination and alignment of components in AI application	"…We must move towards a comprehensive process. You cannot just focus on AI while ignoring other components of PHC—these components must work in harmony." (P30)
		Networking and strengthening organizational participation for effective AI-based health governance	Improving governance through public–private collaboration	"…In governance, AI can be used to strengthen collaboration between public and private sectors—they must work side by side." (P28)
			University participation in AI development and application	"…Universities must contribute. If we aim to use AI for schooling, screening, or referrals, we already have the necessary skills and readiness to apply it." (P30)
			Improving governance and strengthening collaboration between public and private sectors through AI	"…In governance, AI can be used to strengthen cooperation between public and private sectors. When discussing PHC, we must always remember this." (P28)
		Expanding international interactions for AI development	Building effective connections and international collaborations in AI	"…One of our most important requirements, after raising awareness, is creating meaningful links between Iranian society and the international community. The push of technology forces us to move forward." (P20)
	Public Acceptance and Stakeholder Engagement	Raising awareness, public education, and cultural readiness for AI adoption	Strengthening the education system to increase public awareness of AI	"…The education system must reinforce this. For instance, Saudi Arabia mandated its Ministry of Education to increase public awareness of AI. In my opinion, awareness should be strategy number one." (P20)
			Promoting awareness and cultural diffusion among officials and policymakers	"…I am personally working hard in this area—to create a sense of change among universities, officials, and science and technology policymakers." (P20)

			Promoting cultural awareness and education about AI	"…Even if people become convinced, we still need to invest in cultural awareness and education, at least at the theoretical level." (P27)
			Strengthening the education system to increase public awareness of AI	"…The education system must reinforce this. For instance, Saudi Arabia mandated its Ministry of Education to increase public awareness of AI. In my opinion, awareness should be strategy number one." (P20)
			Reducing resistance and improving acceptance of AI use	"…Resistance against this must be reduced so that AI is accepted and given its rightful place." (P21)
		Strengthening social capital and public trust in technological innovation	Strengthening public trust and social capital	"Public trust and creating social capital are crucial, and must be addressed seriously." (P32)
			Enhancing satisfaction and public trust	"…Enhancing satisfaction and public trust in a smart health system can be the foundation. For example, both patients and providers gain opportunities simultaneously, while decision-making and service delivery become more data-driven through AI." (P20)
		Networking and strengthening interdisciplinary expert participation in AI implementation	Stakeholder alliance	"Creating stronger alliances among stakeholders." (P17)
			Networking and coordination among diverse actors to promote AI	"…We face structures and networks of actors at local, national, and international levels—ranging from individuals to systems and various categories. We must orchestrate these actors and networks toward awareness of change." (P20)
			Participation of experts in designing health system programs	"…In the health sector, it seems that experts and thought leaders must sit together to design the national health system program." (P21)
			Creating interdisciplinary teams including legislators, philosophers, and AI experts	"…It is essential to establish interdisciplinary teams. They must include legislators, philosophers, and AI specialists working together to enhance the future of public health." (P28)
			Participation of interdisciplinary experts in AI policymaking	"…Sociologists, behavioral economists, and professional human resource managers should be involved in health policymaking, as they can play a critical role." (P32)
			Leveraging PHC networks to engage people in AI	"…With its broad coverage and strong public trust, the PHC system can serve as a platform to involve citizens in AI processes in meaningful ways." (P25)
Financing and Incentives	Resource allocation and budgeting	Investment and sustainable financing to facilitate AI implementation	Targeted domestic and foreign investments	"Targeted domestic and foreign investments." (P16)
			Influential role of the financing system in facilitating AI adoption by healthcare providers	"…Even if we don't consider the financing system as a driver, it should at least be seen as a highly influential factor in shifting from traditional to modern approaches. If financing organizations demand AI, healthcare providers will certainly move forward more effectively." (P24)
			Reducing costs of AI technologies	"…It is essential to focus specifically on reducing the costs associated with AI technologies and to develop solutions that make their use more economical." (P26)
			Necessity of large-scale investment in AI equipment	"…At the national level, there is a serious need for massive investment in AI equipment, so that the required tools can be provided." (P26)

	Payment Mechanisms	Designing and developing a payment system aligned with AI-based services	Designing new payment models for underserved areas and small communities using AI	"…In communities with fewer than 200,000 residents, we may be able to design and test a new payment model. If successful, it can then be applied in all underserved regions with limited physician availability, helping patients and improving health system management." (P29)
			Designing a mixed payment system	"…We need to design a hybrid model—something like a mix of fixed payments and performance-based incentives, similar to a pay-for-quality approach." (P21)
			Designing a fair payment system to improve provider motivation	"…Payment structures must be designed fairly, so providers have sufficient motivation and do not need to work multiple shifts in different places to make ends meet." (P21)
			Revising tariff systems	"…We must reform the educational and service structures, particularly tariff systems, payment mechanisms, and incentive models, to improve performance." (P21)
Resource Generation	Infrastructure and Technical Equipment	Developing basic infrastructure to support AI implementation	Creating technical infrastructure	"…Hardware has already been acquired, but in the past two years it has become problematic and burdensome." (P22)
			Providing appropriate infrastructure for AI use	"…To optimize the use of AI, proper infrastructure must be established. This includes advanced data analytics systems, integrated information platforms, and reliable communication networks." (P25)
			Strengthening domestic systems by reinforcing infrastructure	"…Instead of full dependence on foreign software, we must strengthen domestic systems, which requires strong local infrastructure." (P26)
			Strengthening and upgrading ICT infrastructure	"…One of the first tasks in the country is to overhaul the entire health system's IT and communication infrastructure, which will enable transformation." (P27)
			Linking with existing infrastructure	"Communication interfaces with the country's existing infrastructure." (P32)
			Need for electricity supply	"Need for electricity supply." (P32)
		Creating smart platforms to enhance AI-based health services	Providing AI infrastructure for mental health and reducing loneliness	"…In mental health, AI could help individuals who are socially isolated, at risk of depression or even suicide. This issue exists in Iran as well as globally." (P20)
			Creating strong and secure infrastructure for AI use in health insurance	"…We must start with health insurance, but first ensure robust infrastructure. Considering risks like hacking, data manipulation, and foreign attacks, security is essential." (P23)
			Developing information infrastructure for data access	"…Developing infrastructure to improve data access and enable AI must be prioritized, especially within health information systems." (P25)
			Need for advanced IT infrastructure and hardware for AI	"…A serious need exists for IT infrastructure and powerful hardware capable of processing and analyzing massive health databases." (P26)
			Creating low-cost data collection infrastructure	"…We need infrastructure that allows the collection of large volumes of data at low cost, otherwise expenses rise significantly." (P18)
			Processing and modeling infrastructure	"Need for processing and modeling infrastructure." (P32)
		Improving software as AI operational platforms	Planning to improve software systems	"…We need a new perspective on software and human resources, with planning that enables the optimal use of AI." (P26)
	Information Resources	Improving the quality, accuracy, and	Accurate and precise data processing management	"Accurate and precise data processing management." (P16)

		reliability of data for AI analysis	Providing correct and reliable data	"First, we must have data. Currently, our data are not reliable." (P22)
			Creating filtering mechanisms for input data	"We lack proper filtering mechanisms to oversee correct data entry by physicians or users." (P22)
			Cleaned datasets	"We need noise-free and purified datasets." (P32)
		Standardization, coordination, and integration of health information systems	Genuine reform of electronic health records	"A genuine reform of electronic health records is required, with strict measures to improve the accuracy and comprehensiveness of health databases." (P17)
			Necessity of coordination and interoperability across different information systems	"…We must connect different information systems, and interoperability between them can be extremely beneficial." (P25)
			Creating coordination and interaction among information systems	"…There must be greater coordination among different information systems, enabling them to interact more effectively." (P25)
			Creating an integrated framework for health data analysis	"…We must link different information systems and move towards an integrated framework capable of analyzing health data effectively. Without such integration, AI cannot be used properly." (P25)
			Creating integration and coordination among various information platforms	"…We must pursue data integration—for example, between systems like SIB, treatment databases, pharmaceutical systems, and insurance systems." (P27)
			Creating interconnections between systems through macro-level policies	"…The interconnection of systems must be mandated through top-level policies." (P27)
			Standardizing data registration processes and preventing incorrect entries	"…We must quickly reform data registration processes at the source, ensuring at the national level that incorrect data are not entered." (P27)
			Developing appropriate data management protocols	"…We must move toward standardizing data entry processes and develop proper protocols for data registration and management." (P27)
			Necessity of establishing an electronic health record network	"…If we establish a proper electronic health record network, such as improving our current SIB system, outcomes will improve." (P25)
			Inter-organizational collaboration for data integration	"Inter-organizational collaboration must be established to achieve data integration." (P22)
			Engaging the private sector in health databases	"The private sector should be involved in structuring health databases and in taking on delegable responsibilities." (P17)
		Knowledge-driven progress in health data analysis with problem-oriented approaches	Creating free datasets for AI research	"…From a policy perspective, datasets must be created and provided freely to students, followed by AI competitions and challenges." (P18)
			Designing AI competitions and challenges for knowledge development	"…The safety debates in language and related issues started in the 1990s and 2000s—we observed competitions then, and now they have evolved into GPTs, ImageNet, and similar algorithms." (P18)
			Need for problem-oriented research and development for AI applications	"…Research and development must be problem-oriented, which is not an easy process. For instance, our experiences with electronic health records revealed many problems that we must learn from." (P30)

			Strengthening scientific foundations in AI applications	"…Our initiatives must have solid scientific foundations." (P31)
			Problem-oriented models	"There is a need for problem-solving models aligned with existing issues (e.g., language models and deep learning models)." (P32)
			Linking theory and practice	"Practice without theory is blind, and theory without practice is futile." (P32)
	Human Resources	Training and employing a specialized workforce for digital health transformation	Developing human resources for effective AI use	"…Most importantly, the hardest part is having people who can work with AI services. We need professionals who can handle restrictions, sanctions, or blocked services. Health services are highly sensitive, and AI provision is still very new." (P19)
			Need for a skilled workforce and necessary technologies for smart health	"…In the new health system, we need skilled human resources, hardware, and software to achieve the aspirations outlined by other experts in this session." (P26)
			Need to train AI specialists	"…Currently, we do not have enough AI experts in the country. We must address this shortage by training more specialized personnel." (P27)
			Training and empowering the workforce to use AI	"…We need to empower our domestic workforce, especially senior AI specialists, who can train and mentor others." (P27)
			Training specialists to use AI	"…Individuals involved in AI must have proper analytical skills to make meaningful contributions." (P21)
			Creating specialized teams free from political affiliations	"…The first priority is to have individuals engaged in AI who are independent of political divisions." (P21)
		Enhancing continuing education for AI readiness in the health system	Advancing AI knowledge and training for physicians, health experts, and policymakers	"…We urgently need to provide training on AI use for physicians, health professionals, and policymakers. Without enhancing their knowledge, effective AI use will not be possible." (P25)
			Need for training and capacity-building in AI use	"…We must pay attention to training and capacity-building in AI use for health experts and policymakers, so they can apply this technology effectively." (P25)
			Necessary AI training programs	"…Therefore, before moving forward, AI education should be embedded in universities to build essential skills." (P30)
			Introductory AI training	"…It would have been beneficial if we had started introductory AI training for health professionals, even at a basic level, as highlighted in the Seventh Development Plan." (P32)
			Designing short-term and targeted AI training	"…We must provide rapid, short-term training for health professionals and related teams, enabling them to use AI more effectively." (P27)
			Reforming educational system and skill development	"…Our educational system must change. Textbooks and references should be updated to sensitize and adapt individuals to new competencies." (P21)
			Revising the educational system based on local needs and environmental conditions	"…We must conduct a comprehensive revision of the education system, tailored to our local environment and conditions." (P21)
		Motivating and preparing	Reforming education, tariffs, and incentive systems to facilitate AI	"…We must reform the educational system, tariff-setting, payment structures, and incentive mechanisms to make AI adoption feasible." (P21)

		organizational actors for technological transformations	Reforming incentive systems for AI facilitation	"…We must reform the educational system, tariff-setting, payment structures, and incentive mechanisms to make AI adoption feasible." (P21)
			The necessity of creating incentives to improve the quality of AI-enabled healthcare services	"…We aim to improve healthcare quality. For instance, insurers should cover costs like streptokinase after strokes; without financial backing, outcomes will fail, even if policies exist." (P23)
			Need to prepare family physicians for adopting new technologies	"…So far, family physicians and frontline providers are among the most prepared to adapt to technological changes." (P23)
			Preserving and strengthening human capital	"…For effective AI implementation, policymakers must prioritize human capital. We are rapidly losing this valuable asset, which is vital for maintaining one of the strongest health systems in the region." (P32)
		Institutional capacity-building and coordination for AI implementation	Need for adequate training of executive staff and leveraging private sector capacities	"…Training must also reach executive staff, while the private sector should provide advisory support." (P23)
			Establishing processes to counter resistance and coordinate across sectors	"…We must create processes to manage resistance across different units—from universities to ministries—when implementing smart health initiatives." (P26)
			Capacity-building and empowerment of the workforce	"Capacity-building and empowerment of the workforce." (P17)
		Continuous evaluation of workforce capacities in the digital era	Assessing skills and competencies required for AI use	"…What is this tool for? If we set a goal in a meeting, do we have the required competencies, skills, and funding to achieve it?" (P31)
